# Synthesis, Anticancer Activities and Molecular Docking Studies of a Novel Class of 2-Phenyl-5,6,7,8-tetrahydroimidazo [1,2-*b*]pyridazine Derivatives Bearing Sulfonamides

**DOI:** 10.3390/molecules27165238

**Published:** 2022-08-17

**Authors:** Otmane Bourzikat, Abdelmoula El Abbouchi, Hamza Ghammaz, Nabil El Brahmi, Elmostfa El Fahime, Arnaud Paris, Richard Daniellou, Franck Suzenet, Gérald Guillaumet, Saïd El Kazzouli

**Affiliations:** 1 Euromed Research Center, Euromed Faculty of Pharmacy, Euromed University of Fes (UEMF), Meknes Road, Fez 30000, Morocco; 2 Institut de Chimie Organique et Analytique, Université d’Orléans, UMR CNRS 7311, BP 6759, CEDEX 2, 45067 Orléans, France; 3 Centre National de la Recherche Scientifique et Technique (CNRST), Angle Avenues des FAR et Allal El Fassi, Hay Ryad, Rabat 10102, Morocco

**Keywords:** 2-phenyl-5,6,7,8-tetrahydroimidazo [1,2-*b*]pyridazine, anticancer activity, sulfonamides, human cancer cell lines

## Abstract

In the present study, new 2-phenyl-5,6,7,8-tetrahydroimidazo [1,2-*b*]pyridazines bearing sulfonamides were synthesized, characterized and evaluated for their anticancer activities. The structures of these derivatives were elucidated by ^1^H NMR, ^13^C NMR, infrared and high-resolution mass spectrometry for further validation of the target compound structures. The anticancer activities of the new molecules were evaluated against five human cancer cell lines, including A-549, Hs-683, MCF-7, SK-MEL-28 and B16-F10 cell lines using 5-fluorouracil and etoposide as the reference drugs. Among the tested compounds, **4e** and **4f** exhibited excellent activities in the same range of the positive controls, 5-fluorouracil and etoposide, against MCF-7 and SK-MEL-28 cancer cell lines, with IC_50_ values ranging from 1 to 10 μM. The molecular docking studies of **4e** and **4f** showed a strong binding with some kinases, which are linked to MCF-7 and SK-MEL-28 cancer cell lines.

## 1. Introduction

Cancer is a complex disease characterized by uncontrolled division and proliferation by blocking the process of normal cell division [1]. It is considered as one of the most serious illnesses that threatens our lives. Moreover, cancer mortality continues to increase and it is expected to surpass cardiovascular mortality in the near future [2]. According to a recent World Health Organization (WHO) global cancer report in 2020, 19.3 million new cases and 10 million deaths have been determined. By 2040, it is projected to have nearly 30.2 million new cancer cases and 16.3 million cancer deaths per year [3]. Therefore, medicinal chemists need to continue designing and developing new anticancer agents with high specificity in order to combat this disease.

Heterocycles are an important class of chemical compounds present in a wide variety of drugs, vitamins, natural products and biomolecules, as well as biologically active molecules [4,5,6,7]. Accordingly, our group has for many years been developing new methods towards the synthesis and functionalization of various heterocyclic systems [8,9,10,11,12,13].

The heterocyclic systems bearing sulfonamide groups have had an important place in medicinal chemistry since Gerhard Domagk discovered their antibacterial activity in 1935 [14]. This discovery has led to breakthroughs in various therapies applied to a variety of diseases [15,16,17,18,19,20,21,22,23]. Notably, some sulfonamide derivatives have been approved by FDA for the treatment of cancer disease [24,25,26,27]. In addition, the hybrid approach has shown to be an important synthetic tool for the preparation of new bioactive molecules from various heterocycles [28].

Protein kinases are a large family of enzymes (518 in human genome) having a crucial rule of catalyzing protein phosphorylation, which is an important mechanism of cells function, such as proliferation, cell cycle, apoptosis, motility, growth and differentiation. Thus, deregulated kinase functions is often linked to cancer spread and development [29].

The computational docking has been used as a powerful strategy for understanding and predicting the molecular interaction of ligands with various biological receptors, such as protein active sites. This interesting protein–ligand interaction can guide the design of molecules and experiments, providing a large set of candidates in medicinal applications. 

In continuation of our research interest in the design and discovery of new anticancer agents [30,31,32,33,34], we aim to report herein the design, synthesis, characterization and anticancer activities of new 2-phenyl-5,6,7,8-tetrahydroimidazo [1,2-*b*]pyridazine derivatives bearing sulfonamides as antiproliferative molecules against five human cancer cell lines, namely A-549 lung cancer, Hs-683 glioma, MCF-7 breast cancer, SKMEL-28 and B16-F10 melanoma cell lines. In order to predict the mechanism of action of the most active compounds, molecular docking studies were also carried out.

## 2. Results

### 2.1. Chemistry

**Scheme 1:**
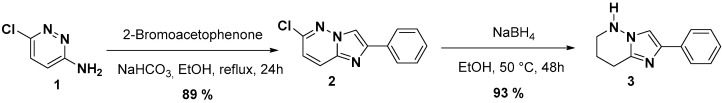
Synthesis of 2-phenyl-5,6,7,8-tetrahydroimidazo [1,2-*b*]pyridazine **3**.

As shown in Scheme 1, the 2-phenyl-5,6,7,8-tetrahydroimidazo [1,2-*b*]pyridazine **3** was synthesized in two steps, starting with a condensation reaction between 3-amino-6-chloropyridazine **1** and 2-bromoacetophenone according to the reported method [35] to obtain 6-chloro-2-phenylimidazo [1,2-*b*]pyridazine **2** in a very good yield (89%) [36]. Compound **2** was then involved in a reduction reaction using eight equivalents of sodium borohydride in ethanol at 50 °C to give product **3** in excellent yield (93%).

To perform the -NH functionalization of the 2-phenyl-5,6,7,8-tetrahydroimidazo [1,2-*b*]pyridazine **3** by sulfonyl moieties, we first studied the sulfonylation reaction in the presence of different bases in order to increase its yield using the model reaction between tosyl chloride and **3** (Table 1). First, we have used two equivalents of tosyl chloride and two equivalents of triethylamine (Et_3_N) in CH_2_Cl_2_. The reaction was stopped after 48 h but the conversion was not complete in leading us to the desired product **4a** in acceptable yield (68%) (entry 1, Table 1). In a second entry, we have used *N,N*-diisopropylethylamine (DIPEA) as a base but we have observed a noticeable drop in the reaction yield to 50% (entry 2, Table 1). We have noticed that the nature of the base plays an important role in this reaction. Surprisingly, when a weak base, such as pyridine, was used, the full conversion was observed after only 1 h with a highly improved yield of 87% (entry 3, Table 1). In the last entry, the decrease in the amount of the tosyl chloride to 1.1 equivalents, respecting the same conditions of entry 3, gave the desired product **4a** with a good yield of 86% (entry 4, Table 1).

**Scheme 2:**
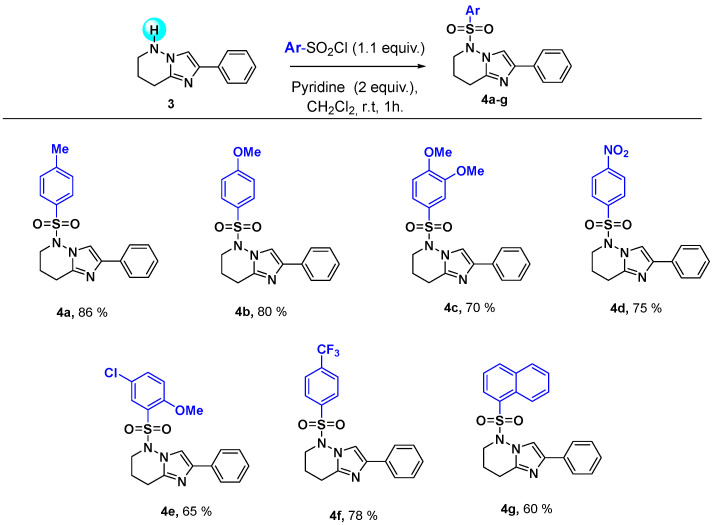
Synthesis and chemical structures of compounds **4a–g**.

Thanks to the optimized reaction conditions, we have synthesized a small chemical library of new 2-phenyl-5,6,7,8-tetrahydroimidazo [1,2-*b*]pyridazine derivatives containing sulfonamides, which was tested on different cancer cell lines. The developed reaction conditions have showed a good compatibility with different aryl sulfonyls bearing various electron-donating or electron-withdrawing groups, such as Cl, OMe, CF_3,_ and NO_2_. The desired products **4a**–**d** and **4f** were obtained with good yields, ranging between 70–86%. In contrast, we have noticed a small decrease in the reaction yields for compounds **4e** and **4g,** which can be explained by the steric hindrance (Scheme 2). All the synthesized compounds were purified by column chromatography and characterized by ^1^H and ^13^C NMR (see Supplementary Materials), infrared and high-resolution mass spectrometry and were in full accordance with their depicted structures.

### 2.2. Antitumor Activity

The synthesized compounds **4a–g** were tested against five human cancer cell lines, namely A549 (lung carcinoma), HS-683 (glioma cancer), MCF-7 (breast carcinoma), and SK-MEL-28 and B16-F1 (melanoma cancer), using MTT (thiazolyl blue tetrazolium bromide, Sigma-Aldrich) assay. The reference drugs were selected to be 5-Fluorouracil (5-FU) and etoposide. The results listed in Table 2 (IC_50_ value, defined as the concentration, inducing a 50% decrease in cell growth after 3 days of incubation), show that the compounds **4e** and **4f** display high inhibitory activities, similar to those obtained with the positive controls 5-FU and etoposide, against cancer cell lines MCF-7 and SK-MEL-28 with IC_50_ values between 9 and 7.8 µΜ, respectively. Moreover, a moderate activity was obtained for compound **4f** against cancer cell lines SK- B16-F1 with IC_50_ value of 10.8 μM. Compounds **4a**, **4c**, **4d** and **4g** demonstrated low activity against cancer cell lines A549, Hs-683, MCF-7 and B16-F1 with IC_50_ values between 10 and 100 μM. Unfortunately, compound **4b** has no activity against the cell line panels tested (IC_50_ > 100 μM).

Structure–activity relationship studies of the prepared compounds **4a–g** toward cancer cell lines have shown that the anticancer activity in compound **4e** against the MCF-7 cell line, increased when electron-donating groups, OMe and Cl, are present at *ortho* and *meta* positions of the phenyl moiety, respectively. In addition, the presence of an electron withdrawing groups CF_3_ attached to the benzene ring at the *para* position, increased the anticancer activity of compound **4f** against SK-MEL-28 cell line. However, the results have revealed that the presence of OMe (an electron-donating group) at the *para* position of the phenyl moiety did not show any inhibitory activity on the cancer cell lines at low concentration. Meanwhile, the membrane–water partition coefficient (cLog P) was calculated using ChemBioDraw Ultra v.12. The compounds **4a–g** have shown clog P between 2.9 and 4.4. These values correlate with those needed to develop drug-like compounds −2 < LogP ≤ 5 according to Lipinski’s rule [37]. 

### 2.3. Molecular Docking Studies

To better elucidate the inhibitory properties of our best compounds (**4e** and **4f**) we have performed a screening of potential kinases implicated in tumorigenesis, based on the 2020 FDA-approved small molecule protein kinase inhibitors (targeting primarily 35 kinases). This screening is performed by a molecular docking approach (see experimental part). 

Molecular docking results have shown a strong binding of **4e** ligand with the following kinases (CSF1R, ErbB2, BRAF and MEK2). It has been reported that high expression of CSF1R is related to breast cancer progression and that high rates of ErbB2 is linked to lower overall survival rates [38,39]. Indeed, the **4e** molecule’s affinity towards the ErbB2 is about (−8.0 Kcal/mol) with 11 non-bond links. Additionally, **4e** was linked to CSF1R with an affinity of (−9.0 Kcal/mol) with 12 non-bond links. Moreover, it has been shown that the expression of BRAF/MEK pathway activity was linked to estrogen-dependent breast cancer [40]. MEK2 and BRAF scored an affinity of −9.1 and −7.9 Kcal/mol, respectively, with 13 non-bond links each (Table 3).

As for the ligand **4f**, the docking data have revealed a high affinity with five kinases (BRAF, CDK4, KIT, MEK2 and PDGFRA). The PDGFRA kinase had the best result with an affinity of −10.5 Kcal/mol, as well as the establishment of 24 non-bond links (2 of them are pi-sulfur bonding type, 15 hydrophobic, 4 hydrogens and 1 halogen bond). The development of certain malignant diseases is associated with the overactivity of PDGF signaling, as well as non-malignant diseases characterized by excessive cell proliferation [41].

BRAF, MEK2, KIT and CDK4 scored an affinity of −9.4, −9.1, −9.1, −9.6 and a total non-bond of 17, 19, 19 and 17, respectively. Previous studies have shown a direct link between skin cancer and mutated BRAF kinase accounting for 50% of total melanomas [42], decreasing its expression could benefit in controlling the cancer. Although MEK2 and BRAF kinase are part of the same MAPK signaling pathway, MEK2, however, has not yet been shown to possess any effect on the melanoma pathogenesis [43]. KIT plays an important role in the activation of many signaling pathways, including the MAPK pathway. Consequently, it contributes in what is known as KIT-derived melanoma [44]. CDK4 appears in 90% of melanoma cases; furthermore, MAPK signaling pathway upregulates D1 cycling expression, resulting in CDK4 pathway enhancement [45]. These results have shown that the **4f** molecule can be considered as potential skin cancer treatment. 

## 3. Materials and Methods

### 3.1. General Procedures

All chemicals, starting materials and solvents used in this study were bought from Fluorochem, Sigma-Aldrich or Alfa Aesar and used without further purification. All reactions were carried out under regular conditions. All solvents were obtained from commercial sources and were used as received. The evolution of the reactions was monitored by thin layer chromatography (TLC) on aluminum sheets covered with Merck 60 F254 silica gel (thickness 0.2 mm), and the revelation was carried out under ultraviolet lamp regulated at 254 nm. Column chromatography was performed on silica gel Merck 60 μm (215–400 mesh). Melting points (mp [°C]) were carried out by open capillary tubes and are uncorrected using Thermo scientific digital melting point IA9200. The NMR spectra were recorded on a Bruker AC 300 or 400 MHz instruments at room temperature, using TMS as the internal standard and chloroform-d as a solvent. Chemical shifts (δ) are measured in parts per million (ppm). Infrared spectra were recorded on a Thermo Scientific, Nicolet IS50 FT-IR. High-resolution mass spectra (HRMS) was performed on a Maxis Bruker 4G.

### 3.2. Synthesis and Characterization

*6-Chloro-2-phenylimidazo [1,2-b]pyridazine (**2**).* The 6-chloro-2-phenylimidazo [1,2-*b*]pyridazine (**2**) was prepared as described in the literature [35,36]. ^1^H NMR (300 MHz, CDCl_3_): δ = 8.19 (s, 1H), 7.95–7.91 (m, 2H), 7.87 (d, *J* = 9.0 Hz, 1H), 7.47–7.35 (m, 3H), 7.02 (d, *J* = 9.0 Hz, 1H). ^13^C NMR (75 MHz, CDCl_3_): δ = 146.8, 133.1, 132.9, 129.9 (2*CH), 128.8, 126.4, 126.1 (2*CH), 123.0, 119.0, 113.2. IR: ν (cm^−1^) = 3065 (C–H), 1673 (C=C), 1516 (C=N), 1446 (C=N), 1100 (C–N) 826, 801(C–C), 685 (C–Cl).

*2-Phenyl-5,6,7,8-tetrahydroimidazo [1,2-b]pyridazine (**3**).* To a solution of compound **(2)** (1.00 g, 4.36 mmol) in ethanol (20 mL), sodium borohydride (0.99 g, 26.19 mmol) was added and the mixture was stirred at 50 °C for 48 h. The reaction was quenched with drops of water, then the solvent was removed under reduced pressure. The residue was dissolved in CH_2_Cl_2_ (20 mL), washed three times with water and the organic phase was separated, dried over magnesium sulfate and evaporated to dryness to afford compound **(3)** as a white solid (yield 93%). Mp: 118–119 °C. ^1^H NMR (300 MHz, CDCl_3_): δ 7.70 (dd, *J* = 6.0 Hz, 3.0 Hz, 2H), 7.35 (dd, *J* = 9.0 Hz, 6.0 Hz, 2H), 7.21 (dd, *J* = 6.0 Hz, 9.0 Hz, 2H), 7.08 (s, 1H), 3.24–3.20 (m, 2H), 2.96–2.88 (m, 2H), 1.94–1.86 (m, 2H). ^13^C NMR (75 MHz, CDCl_3_): δ 139.9, 137.3, 134.4, 128.6 (2*CH), 126.6, 124.5 (2*CH), 113.1, 45.0, 22.3, 22.2. IR: ν (cm^−1^) = 3165 (N–H), 2954(C–H), 1600 (C=C), 1443 (C=N), 1070 (C–N), 755 (C–C). HRMS (ESI): *m/z* calcd for C_12_H_14_N_3_ [M + H]^+^ 200.1182; found: 200.1182.

General procedure for the synthesis of compounds (**4a–g**).

Compound **(3)** (0.20 g, 0.50 mmol) was dissolved in CH_2_Cl_2_ (6 mL), then pyridine (0.08 g, 1.00 mmol) was added. After 5 min of stirring, sulfonyl chloride (0.55 mmol) was added and the reaction mixture was stirred at room temperature for 1 h. After evaporation, the medium was extracted with CH_2_Cl_2_, and the organic phase was washed with water (3 × 10 mL), dried over anhydrous MgSO_4_ and then concentrated under reduced pressure. The obtained products were purified by silica gel chromatography to give **4a–g** as a solid. 

*5-((4-Methyphenyl)sulfonyl)-2-phenyl-5,6,7,8-tetrahydroimidazo [1,2-b]pyridazine (**4a**).* Compound **(4a)** was prepared according to the general procedure; the desired product was obtained after chromatography on silica gel (eluent: DCM/EtOAc 7/3) as a pale yellow solid (86%). Mp: 143–144 °C. ^1^H NMR (300 MHz, CDCl_3_): δ 7.78 (dt, *J* = 8.2, 1.7 Hz, 2H), 7.55 (s, 1H), 7.48–7.36 (m, 4H), 7.33–7.27 (m, 3H), 3.99–3.92 (m, 2H), 2.65 (t, *J* = 7.2 Hz, 2H), 2.43 (s, 3H), 1.60 (qd, *J* = 7.2, 4.4 Hz, 2H). ^13^C NMR (75 MHz, CDCl_3_): δ 146.1, 140.6, 137.3, 132.5, 130.4 (2*CH), 128.7 (2*CH), 128.1 (2*CH), 127.5, 125.2, 124.9 (2*CH), 114.7, 47.5, 21.7, 20.6, 16.8. IR: ν (cm^−1^) = 2358 (C–H), 1362 (S=O asymmetrical), 1166 (S=O symmetrical), 1067 (C–N), 998 (C–C). HRMS (ESI): *m/z* calcd for C_19_H_20_N_3_O_2_S [M + H]^+^ 354.1272; found: 354.1270.

*5-((4-Methoxyphenyl)sulfonyl)-2-phenyl-5,6,7,8-tetrahydroimidazo [1,2-b]pyridazine (**4b**).* Compound **(4b)** was prepared according to the general procedure; the desired product was obtained after chromatography on silica gel (eluent: DCM/EtOAc 6/4) as a brown solid (80%). Mp: 142–143 °C. ^1^H NMR (400 MHz, CDCl_3_): δ 7.80–7.72 (m, 2H), 7.53 (s, 1H), 7.50–7.44 (m, 2H), 7.39 (ddd, *J* = 9.3, 5.9, 1.7 Hz, 2H), 7.37–7.27 (m, 1H), 6.95–6.91 (m, 2H), 3.97–3.91 (m, 2H), 3.85 (s, 3H), 2.61 (t, *J* = 7.2 Hz, 2H), 1.68–1.57 (m, 2H). ^13^C NMR (101 MHz, CDCl_3_): δ 164.4, 140.4, 138.1, 133.4, 130.4 (2*CH), 128.6 (2*CH), 127.1, 126.9, 124.7 (2*CH), 114.8 (2*CH), 114.7, 55.7, 47.5, 20.9, 17.1. IR: ν (cm^−1^) = 2952 (C–H), 1592 (C=C), 1362 (S=O asymmetrical), 1160 (S=O symmetrical), 1070 (C–O). HRMS (ESI): m/z calcd for C_19_H_20_N_3_O_3_S [M + H]^+^ 370.1226; found: 370.1219.

*5-((3,4-Dimethoxyphenyl)sulfonyl)-2-phenyl-5,6,7,8-tetrahydroimidazo [1,2-b]pyridazine (**4c**).* Compound **(4c)** was prepared according to the general procedure; the desired product was obtained after chromatography on silica gel (eluent: DCM/EtOAc 6/4) as a brown solid (70%). Mp: 132–133 °C. ^1^H NMR (400 MHz, CDCl_3_): δ 7.78–7.71 (m, 2H), 7.59 (t, *J* = 1.6 Hz, 1H), 7.41–7.34 (m, 2H), 7.27 (d, *J* = 2.1 Hz, 1H), 7.26–7.23 (m, 1H), 6.92 (t, *J* = 5.9 Hz, 1H), 6.75 (d, *J* = 2.1 Hz, 1H), 3.99–3.94 (m, 2H), 3.93 (t, *J* = 2.4 Hz, 3H), 3.66 (t, *J* = 1.8 Hz, 3H), 2.65–2.58 (m, 2H), 1.65–1.55 (m, 2H). ^13^C NMR (101 MHz, CDCl_3_): δ 154.1, 149.4, 140.5, 138.0, 133.2, 128.6 (2*CH), 127.2, 126.8, 124.7 (2*CH), 122.1, 114.7, 110.9, 109.7, 56.2, 56.2, 47.6, 21.0, 16.9. IR ν (cm^−1^) = 2359 (C-H), 1585 (C=C), 1508 (C=C), 1361 (S=O asymmetrical), 1263 (C–O), 1159 (S=O symmetrical), 1013 (C–N). HRMS (ESI): *m/z* calcd for C_20_H_22_N_3_O_4_S [M + H]^+^ 400.1324; found: 400.1325.

*5-((4-Nitrophenyl)sulfonyl)-2-phenyl-5,6,7,8-tetrahydroimidazo [1,2-b] pyridazine (**4d**).* Compound **(4d)** was prepared according to the general procedure; the desired product was obtained after chromatography on silica gel (eluent: DCM/EtOAc 6/4) as a brown solid (75%). Mp: 218–219 °C. ^1^H NMR (400 MHz, CDCl_3_): δ 8.37–8.29 (m, 2H), 7.80–7.73 (m, 4H), 7.55 (s, 1H), 7.43–7.37 (m, 2H), 7.32–7.27 (m, 1H), 4.06–3.98 (m, 2H), 2.65 (t, *J* = 7.2 Hz, 2H), 1.67–1.54 (m, 2H). ^13^C NMR (101 MHz, CDCl_3_): δ 151.0, 141.7, 140.0, 138.8, 133.0, 129.5 (2*CH), 128.7 (2*CH), 127.4, 124.8 (2*CH), 124.7 (2*CH), 114.1, 47.9, 21.0, 17.6. IR: ν (cm^−1^) = 2359 (C–H), 1529 (C=C), 1346 (S=O asymmetrical), 1168 (S=O symmetrical), 1066 (N–O), 1002 (C–C). HRMS (ESI): *m/z* calcd for C_18_H_17_N_4_O_4_S [M + H]^+^ 385.0973; found: 385.0965.

*5-((5-Chloro-2-methoxyphenyl)sulfonyl)-2-phenyl-5,6,7,8-tetrahydroimidazo [1,2-b]pyridazine (**4e**).* Compound **(4e)** was prepared according to the general procedure; the desired product was obtained after chromatography on silica gel (eluent: DCM/EtOAc 7/3) as a brown solid (65%). Mp: 135–136 °C. ^1^H NMR (400 MHz, CDCl_3_): δ 7.94 (d, *J* = 2.6 Hz, 1H), 7.79–7.72 (m, 2H), 7.59–7.51 (m, 2H), 7.38 (t, *J* = 7.7 Hz, 2H), 7.29–7.21 (m, 1H), 6.88 (d, *J* = 8.9 Hz, 1H), 4.00–3.93 (m, 2H), 3.51 (s, 3H), 2.82 (t, *J* = 7.2 Hz, 2H), 1.76–1.66 (m, 2H).^13^C NMR (101 MHz, CDCl_3_): δ 156.4, 139.9, 137.4, 136.0, 133.7, 131.3, 128.6 (2*CH), 126.9, 126.9, 125.8, 124.7 (2*CH), 115.1, 113.9, 56.7, 47.6, 21.2, 17.7. IR: ν (cm^−1^) = 2939 (C–H), 1479 (C=C), 1277 (S=O asymmetrical), 1159 (S=O symmetrical), 1004 (C–C). HRMS (ESI): m/z calcd for C_19_H_19_ClN_3_O_3_S [M + H]^+^ 404.0828; found: 404.0830.

*5-((4-(Trifluoromethyl)phenyl)sulfonyl)-2-phenyl-5,6,7,8-tetrahydroimidazo [1,2-b]pyridazine (**4f**)*. Compound (**4f**) was prepared according to the general procedure; the desired product was obtained after chromatography on silica gel (eluent: DCM/EtOAc 7/3) as a white solid (78%). Mp: 157–158 °C. ^1^H NMR (300 MHz, CDCl_3_): δ 7.77 (d, *J* = 8.2 Hz, 4H), 7.70 (d, *J* = 8.3 Hz, 2H), 7.55 (s, 1H), 7.40 (dd, *J* = 10.5, 4.8 Hz, 2H), 7.29 (t, *J* = 7.4 Hz, 1H), 4.07–3.89 (m, 2H), 2.62 (t, *J* = 7.2 Hz, 2H), 1.71–1.52 (m, 2H). ^13^C NMR (75 MHz, CDCl_3_): δ 140.2, 139.5, 138.5, 136.1 (q, *J*_Cq-F_ = 33.4 Hz, C_q_-F), 133.0, 128.7, 128.6, 127.4 (2*CH), 126.8 (q, ^3^*J*_CHAr-F_ = 3.7 Hz, 2C, CHAr), 124.8 (2*CH), 122.8 (q, ^1^*J*_C-F_ = 273.3 Hz, CF_3_), 114.2 (2*CH), 47.8, 20.9, 17.5. ^19^F NMR (376 MHz, CDCl_3_): δ −63.2 (s, 1F). IR: ν (cm^−1^): 3167 (C–H), 1612 (C=C), 1372 (S=O asymmetrical), 1324 (S=O symmetrical), 1061 (C–N). HRMS (ESI): *m/z* calcd for C_19_H_17_F_3_N_3_O_2_S [M + H]^+^ 408.0989; found: 408.0988.

*5-(Naphthalen-1-ylsulfonyl)-2-phenyl-5,6,7,8-tetrahydroimidazo [1,2-b]pyridazine (**4g**).* Compound **(4g)** was prepared according to the general procedure; the desired product was obtained after chromatography on silica gel (eluent: DCM/EtOAc 7/3) as a white solid (60%). Mp: 79–80 °C. ^1^H NMR (300 MHz, CDCl_3_): δ 8.34 (d, *J* = 8.7 Hz, 1H), 8.20 (dt, *J* = 8.3, 1.9 Hz, 2H), 7.96 (d, *J* = 7.7 Hz, 1H), 7.64–7.52 (m, 5H), 7.37–7.30 (m, 2H), 7.23 (ddd, *J* = 6.7, 4.0, 1.2 Hz, 1H), 6.89 (s, 1H), 4.07–3.95 (m, 2H), 2.63 (t, *J* = 7.3 Hz, 2H), 1.79–1.66 (m, 2H). ^13^C NMR (75 MHz, CDCl_3_): δ 141.1, 138.1, 136.2, 134.2, 133.5, 132.2, 131.7, 129.0, 128.9, 128.7, 128.5 (2*CH), 127.5, 127.0, 124.8 (2*CH), 124.3 (2*CH), 114.1, 47.1, 20.7, 18.1. IR: ν (cm^−1^): 1609 (C=C), 1505 (C=C), 1368 (S=O asymmetrical), 1166 (S=O symmetrical). HRMS (ESI): *m*/*z* calcd for C_22_H_20_N_3_O_2_S [M + H]^+^ 390.1277; found: 390.1270.

### 3.3. Biology

The growth level of four cancer cell lines was determined using a colorimetric MTT (thiazolyl blue tetrazolium bromide, Sigma, Saint-Quentin-Fallavier Cedex, France) assay. Cancer cell lines and growth medium were obtained from CLS Cell Line Service GmbH, Eppelheim, Germany). Human skin melanoma SK-MEL-28, mouse melanoma skin B16-F1 and human brain glioma HS683 were grown in DMEM, supplemented with 4.5 g/L glucose, 2 mM L-glutamine and 10% FBS. The human lung carcinoma cell line A-549 was grown in DMEM: Ham’s F12 (1:1) supplemented with 2 mM L-glutamine and 5% FBS and human breast adenoma carcinoma MCF-7 in EMEM supplemented with 2 mM L-glutamine, sodium pyruvate, NEAA, 10 µg/mL insulin human and 10% FBS. MTT assay is based on the reduction of the yellow product thiazolyl blue tetrazolium bromide (MTT) to purple-blue formazan by mitochondrial dehydrogenase of metabolically active cells. The number of living cells after incubation in the presence (or absence, control) of the tested molecule is directly proportional to the blue color, which was measured by spectrophotometry. Briefly, cells were seeded (100 µL of a 2,5.10^4^ cells/mL suspension) in 96-well culture plates (Nunc™ Edge 2.0, Fisher, Illkirch Cedex, France) and incubated for 24 h. Each compound (starting from DMSO solutions, stable for months) was assessed in serial dilution (four concentrations in 0.1% DMSO at the highest concentration) in three replicates (n = 3) and incubated for 72 h. Thereafter, MTT (5 mg/mL solution in PBS) was added to each well (10% *v*/*v*) and cells were further incubated for 4 h. Then, after removing the culture medium, the blue crystals were dissolved in 100 µL 100% DMSO and absorbance measured at 540 nm using a 620 nm reference. Absorbance of the serial dilution of each cell line treated under the same conditions, but without the tested compounds, was measured to generate a standard curve, allowing IC_50_ determination (IC_50_ is defined as the concentration reducing cell growth by 50%).

### 3.4. Molecular Docking

The approach starts by retrieving the PDB structure of the candidate kinases from RSCB PDB database. Discovery Studio software (V 21.0.20298) was used to prepare the downloaded molecules by removing water molecules, already existent ligands and adding polar hydrogens. In the next step we used AutoDockTools-1.5.6 to convert PDB structure of the prepared macromolecule to PDBQT format after adding Kollman, Gasteiger charges and AD4 type atoms. The ligand **4f** was energy minimized using open babel binaries (2.4.0 release) and converted to PDBQT format using AutoDockTools-1.5.6. Vina executable was run after specifying X, Y and Z coordinates of the macromolecule, as well as the radius value in the configuration file. The resulting conformations were separated using vina split executable and visualized in the Discovery Studio software.

## 4. Conclusions

In conclusion, a new series of 2-phenyl-5,6,7,8-tetrahydroimidazo [1,2-*b*]pyridazine derivatives bearing sulfonamides were designed and synthesized in a three-step sequence, starting with a condensation reaction between 2-bromoacetophenone and 3-amino-6-chloropyridazine. Then, the obtained products were subjected to a reduction reaction. The final step, the *N*-sulfonylation reaction, was achieved by using a variety of sulfonyl chlorides. The in vitro cytotoxic potential of these new compounds were screened against A549, HS-683, MCF-7, SK-MEL-28 and B16-F1 cell lines. The compounds **4e** and **4f** displayed good cytotoxic activities against cancer cell lines, MCF-7 and SK-MEL-28, with IC_50_ values in low micromolar range. Molecular docking studies of the **4e** have showed a strong binding with CSF1R, ErbB2, BRAF and MEK2 kinases. On the other hand, the compound **4f** also presents high binding affinities with BRAF, CDK4, KIT, MEK2 and PDGFRA kinases. Importantly, the overexpression of these kinases is linked to MCF-7 and SK-MEL-28 cancer cell line proliferations. The obtained results are very promising and demonstrate that the new compounds **4e** and **4f** could lead towards anticancer drug development.

## Figures and Tables

**Table 1:** Optimization of the conditions of the sulfonylation reaction.

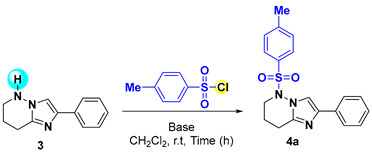				
Entry	Tosyl Chloride (Equiv.)	Base (2 Equiv.)	Time (h)	Yield%
1	2	Et_3_N	48	68
2	2	DIPEA	48	55
3	2	Pyridine	1	87
4	1.1	Pyridine	1	86

**Table 2:** The cytotoxic activities of the synthesized compounds and standard anticancer agents expressed in terms of IC_50_ (μM).

Compounds	Human Cancer Cell Lines IC_50_ (μM) ^a^					
	A549 ^b^	HS-683 ^c^	MCF-7 ^d^	SK-MEL-28 ^e^	B16-F1 ^f^	c Log P ^g^
4a	10–100	>100	>100	>100	10–100	3.7
4b	>100	>100	>100	>100	>100	3.1
4c	10–100	>100	10–100	>100	>100	2.9
4d	>100	10–100	10–100	>100	10–100	3.0
4e	>100	>100	9.4	>100	>100	4.0
4f	>100	67.9	96.5	7.8	10.8	4.1
4g	>100	>100	>100	10–50	>100	4.4
5-FU	1.2	4.3	2.3	3.3	0.3	
Etoposide	0.9	0.8	3.3	1.1	1.3	

^a^ IC_50_ values (μM): Drug concentration responsible for the inhibition of 50% of the growth of the specified cell line after 72 h. ^b^ Human lung cancer cell line. ^c^ Human glioma cancer cell line. ^d^ Human breast cancer cell line. ^e^ Human melanoma cancer cell line. ^f^ Mouse melanoma cancer cell line. ^g^ Partition coefficient, a measure of lipophilicity.

**Table 3:** Illustrations in 3D and 2D of the best interactions for **4e** (A) and **4f** (B) and aminos acids within the active site of different kinases.

**4e**		
BRAF	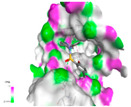	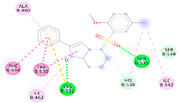
CSF1R	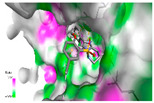	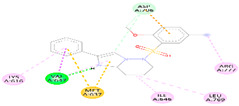
ErbB2	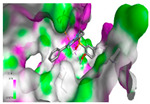	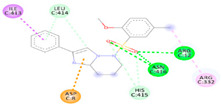
MEK2	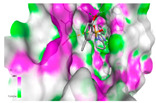	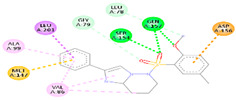
**4f**		
	**Pocket**	**2D diagram**
PDGFRA	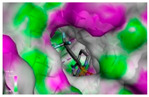	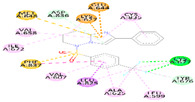
BRAF	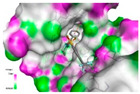	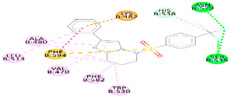
MEK2	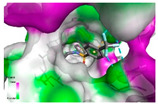	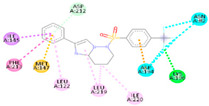
KIT	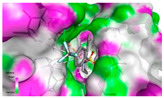	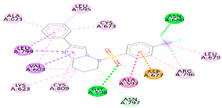
CDK4	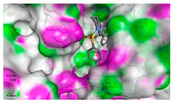	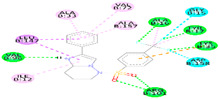

## Supplementary Materials

The following supporting information can be downloaded at: https://www.mdpi.com/article/10.3390/molecules27165238/s1, ^1^H and ^13^C NMR spectra and HRMS spectra.

## Sample Availability

Samples of the compounds **4a–g** are available from the authors.
