# Supplementary material for: Synthesis, Anticancer Activities and Molecular Docking Studies of a Novel Class of 2-Phenyl-5,6,7,8-tetrahydroimidazo [1,2-b]pyridazine Derivatives Bearing Sulfonamides
Source: Molecules. 2022 Aug 17;27(16):5238. doi: 10.3390/molecules27165238 (PMC9416205; doi:10.3390/molecules27165238)

Supplementary

Synthesis, Anticancer Activities and Molecular Docking Studies of a Novel Class of 2-Phenyl-5,6,7,8-tetrahydroimidazo [1,2-*b*]pyridazine Derivatives Bearing Sulfonamides

Otmane Bourzikat [1,2], Abdelmoula El Abbouchi [1], Hamza Ghammaz [3], Nabil El Brahmi [1], Elmostfa El Fahime [1,3], Arnaud Paris [2], Richard Daniellou [2], Franck Suzenet [2], Gérald Guillaumet [1,2] and Saïd El Kazzouli [1,*]

[1] Euromed Research Center, Euromed Faculty of Pharmacy, Euromed University of Fes (UEMF), Meknes Road, Fez 30000, Morocco
[2] Institut de Chimie Organique et Analytique, Université d'Orléans, UMR CNRS 7311, BP 6759, CEDEX 2, 45067 Orléans, France
[3] Centre National de la Recherche Scientifique et Technique (CNRST), Angle Avenues des FAR et Allal El Fassi, Hay Ryad, Rabat 10102, Morocco
* Correspondence: s.elkazzouli@ueuromed.org; Tel.: +212-661299565

Contents

^{1}H and ^{13}C NMR spectra for all final compounds 2, 3 and 4a-g ………………………………………S2

HRMS spectra for all final products 3 and 4a-g ……………………………………………………………S12

NMR (^{1}H, ^{13}C) SPECTRA:

6-chloro-2-phenylimidazo[1,2-b]pyridazine (2)

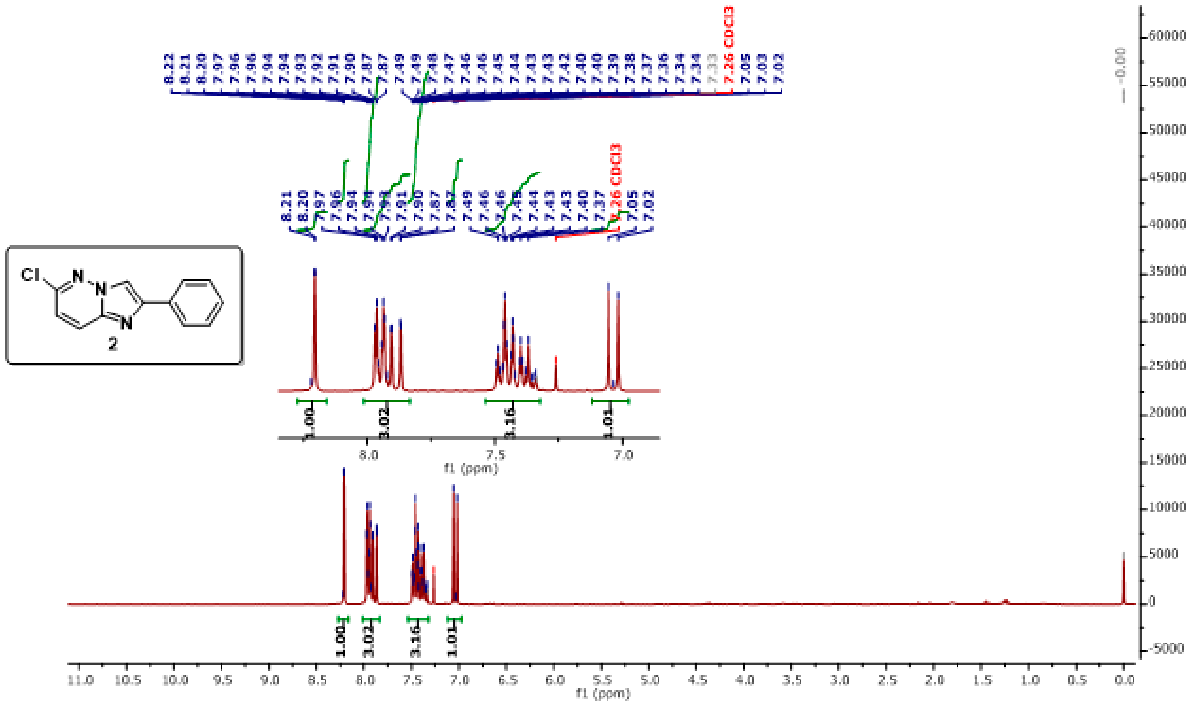

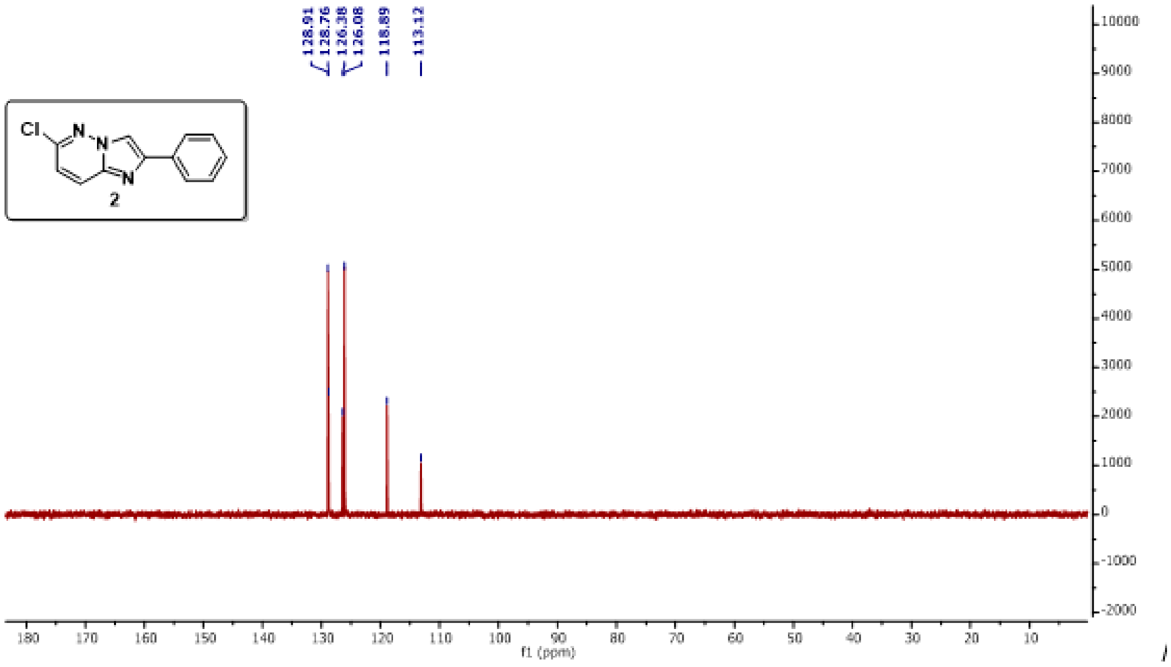

2-phenyl-5,6,7,8-tetrahydroimidazo[1,2-b]pyridazine (3)

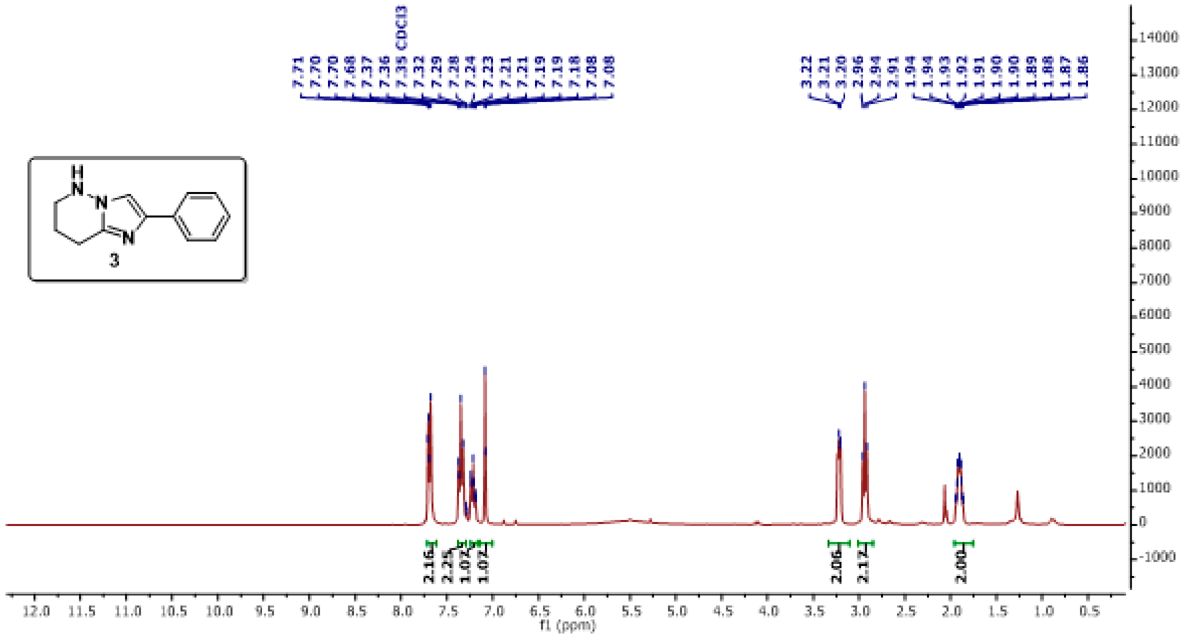

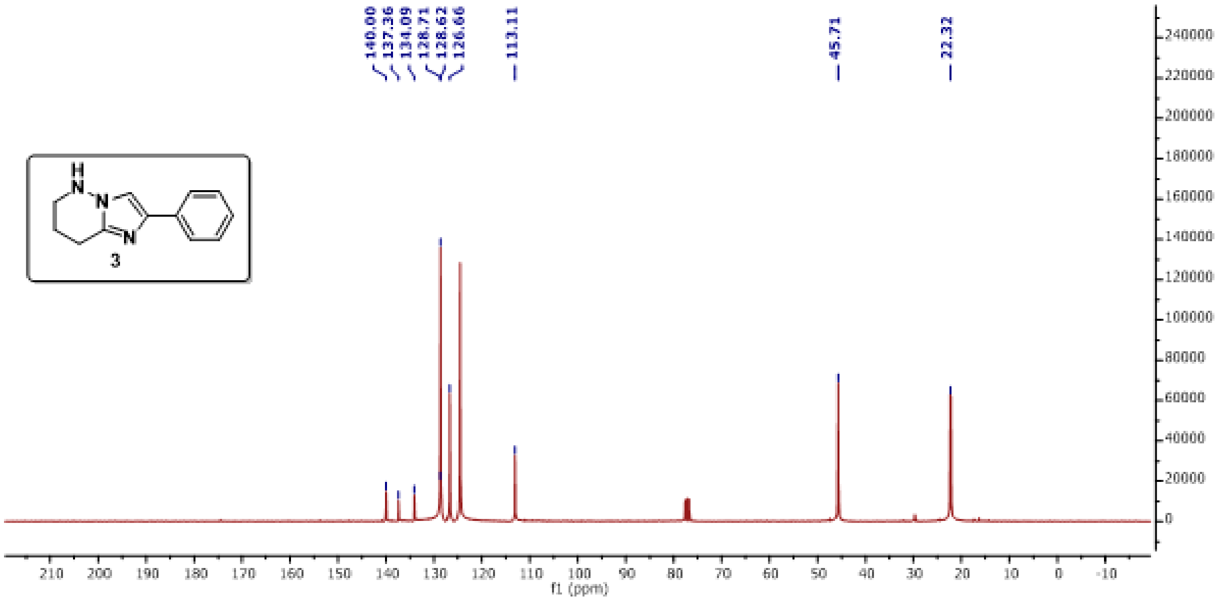

5-((4-methyphenyl)sulfonyl)-2-phenyl-5,6,7,8-tetrahydroimidazo[1,2-b]pyridazine (4a)

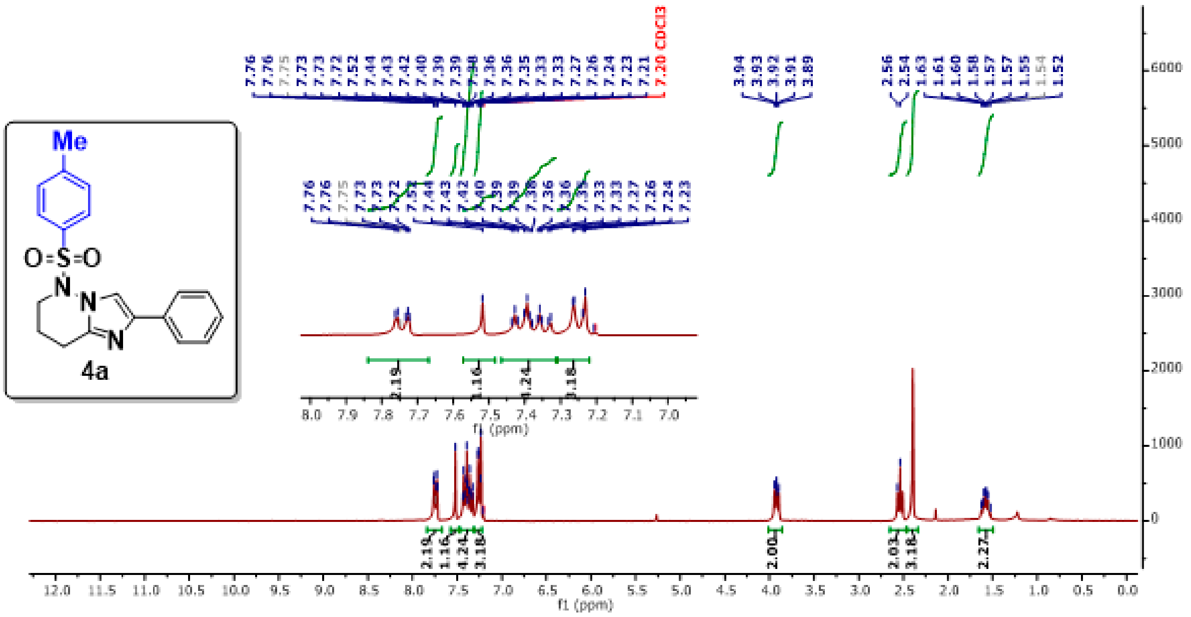

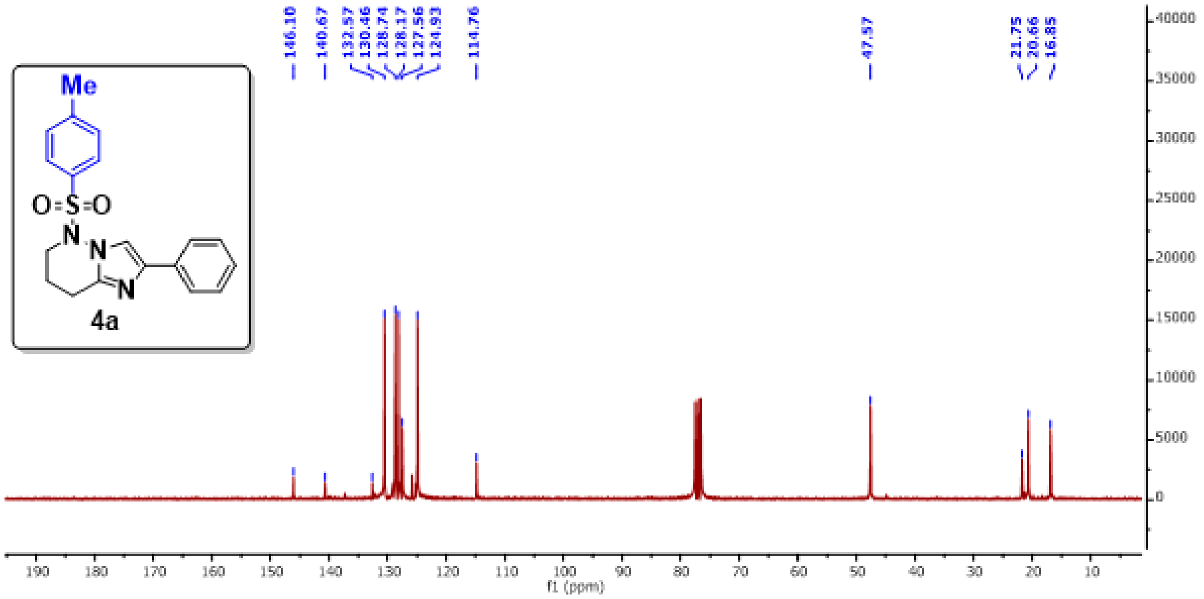

5-((4-methoxyphenyl)sulfonyl)-2-phenyl-5,6,7,8-tetrahydroimidazo[1,2-b]pyridazine (4b)

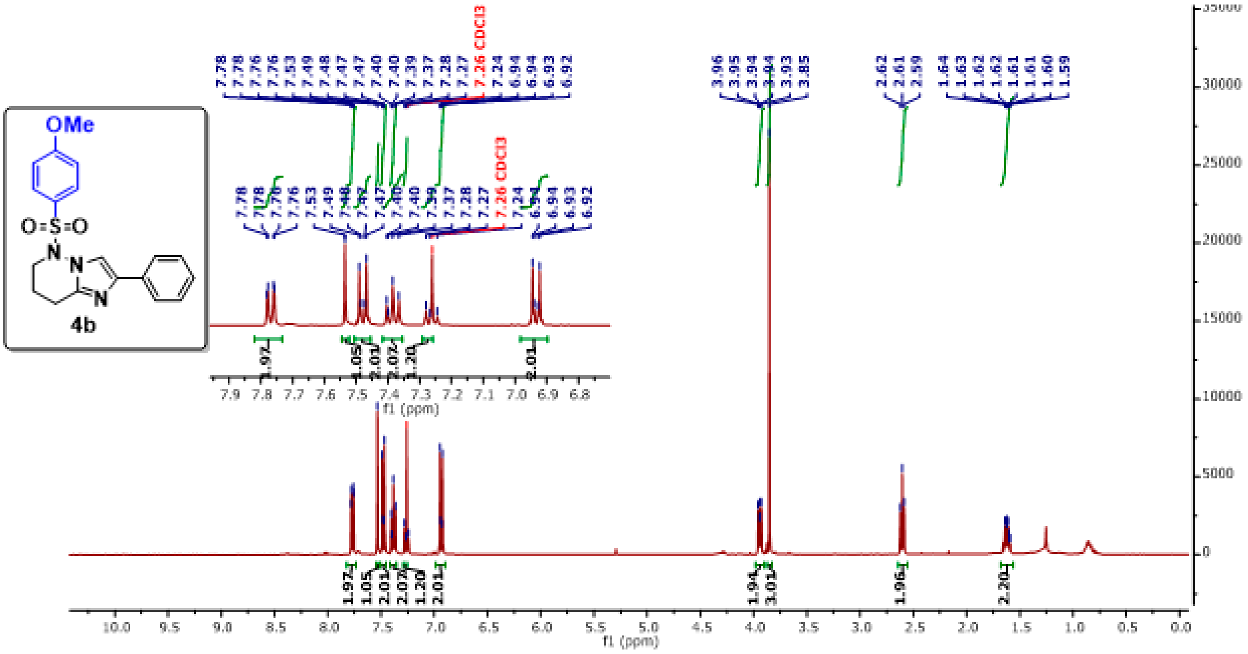

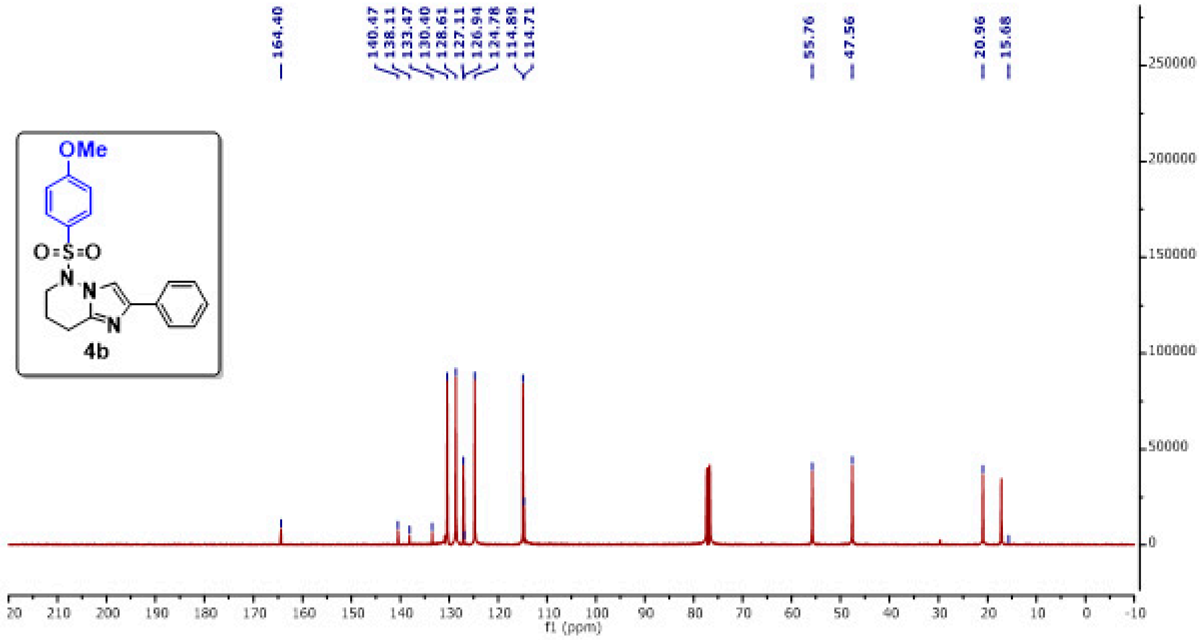

5-((3,4-dimethoxyphenyl)sulfonyl)-2-phenyl-5,6,7,8-tetrahydroimidazo[1,2-b]pyridazine (4c)

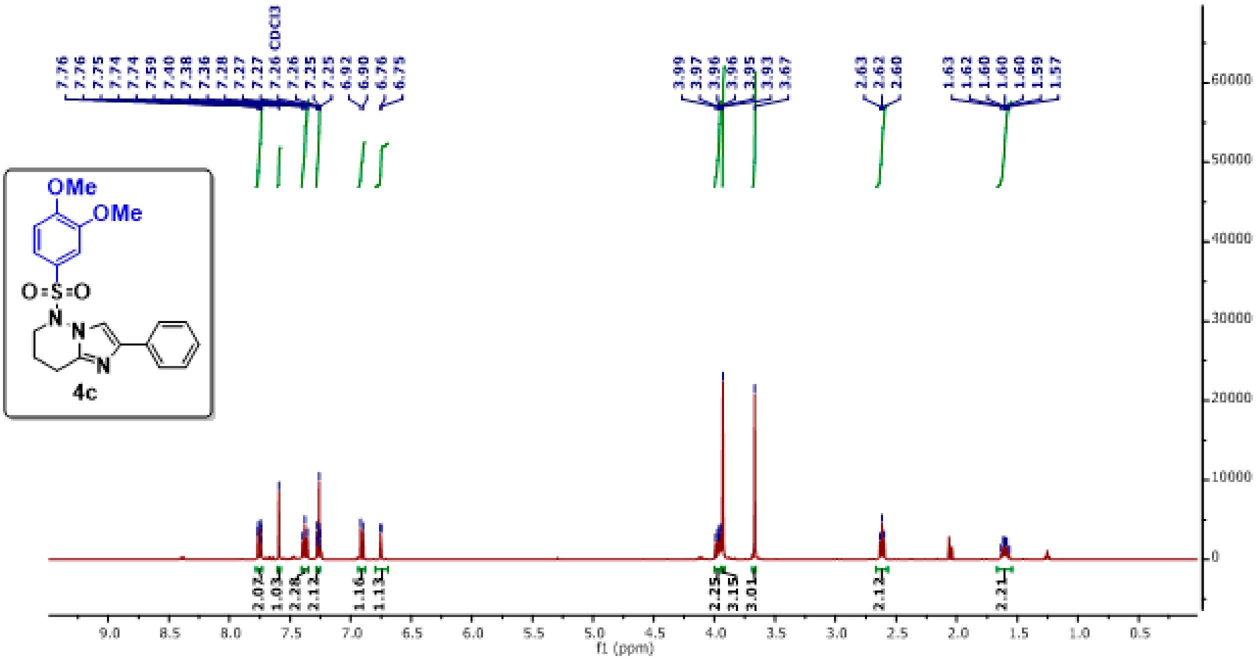

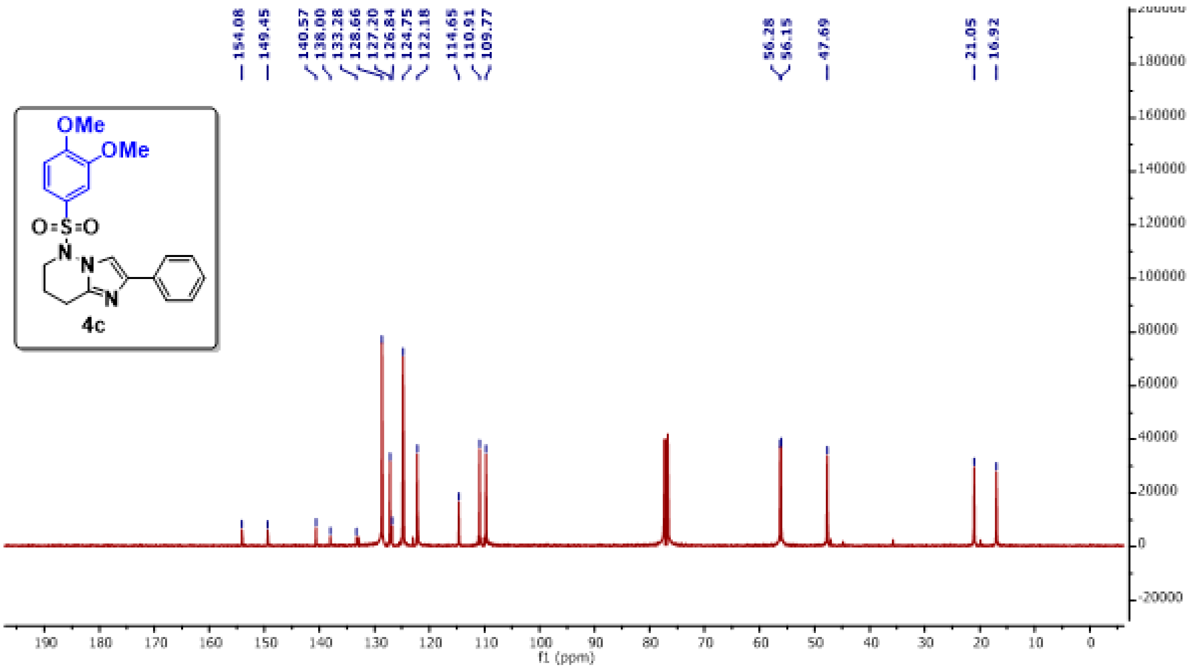

5-((4-nitrophenyl)sulfonyl)-2-phenyl-5,6,7,8-tetrahydroimidazo[1,2-b] pyridazine (4d)

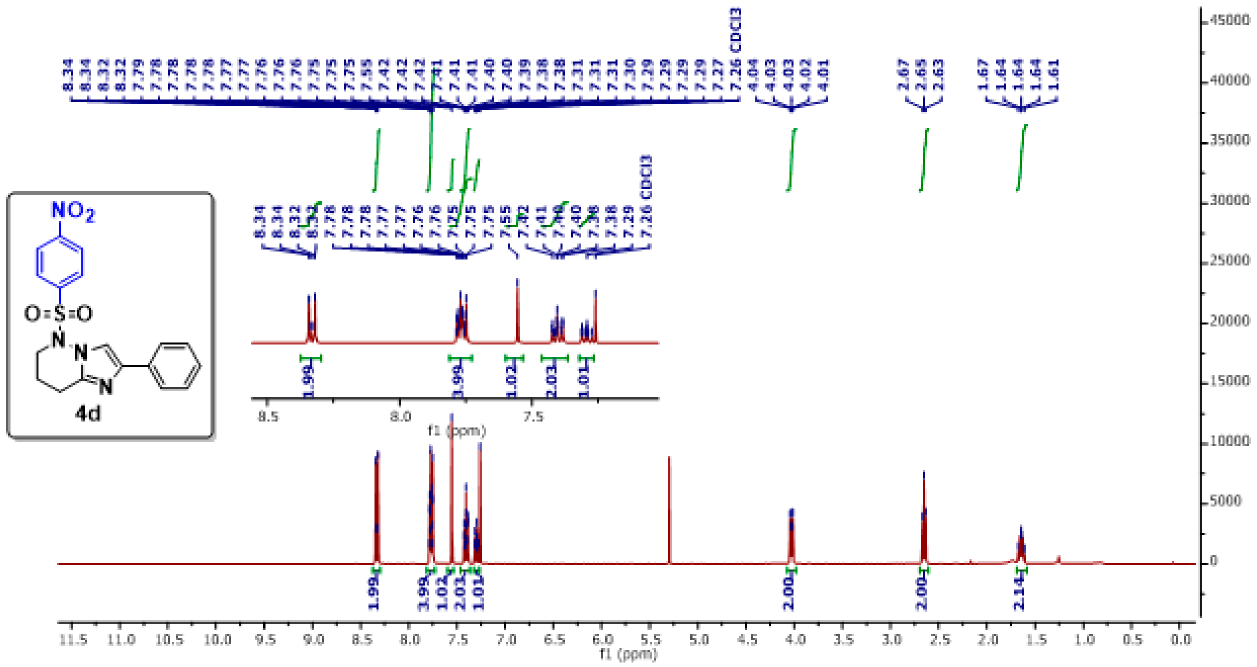

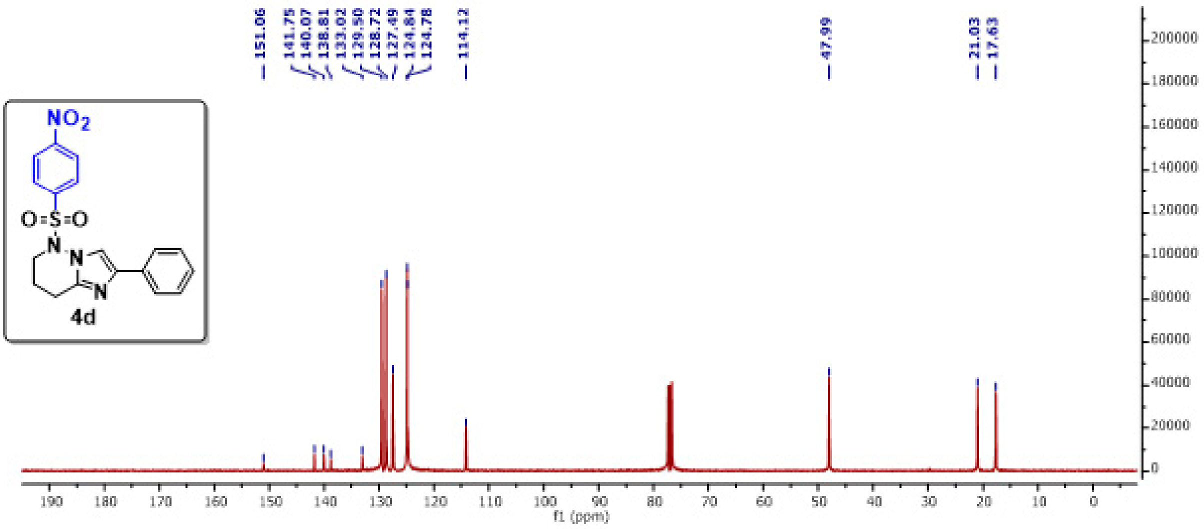

5-((5-chloro-2-methoxyphenyl)sulfonyl)-2-phenyl-5,6,7 ,8-tetrahydroimidazo[1,2-b]pyridazine (4e)

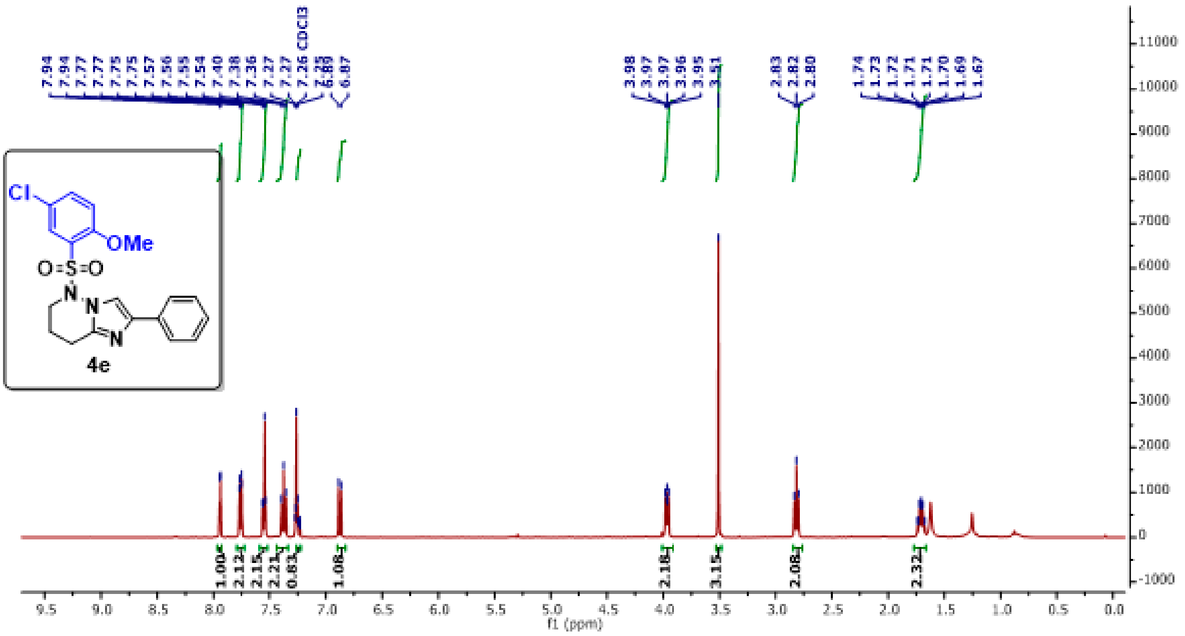

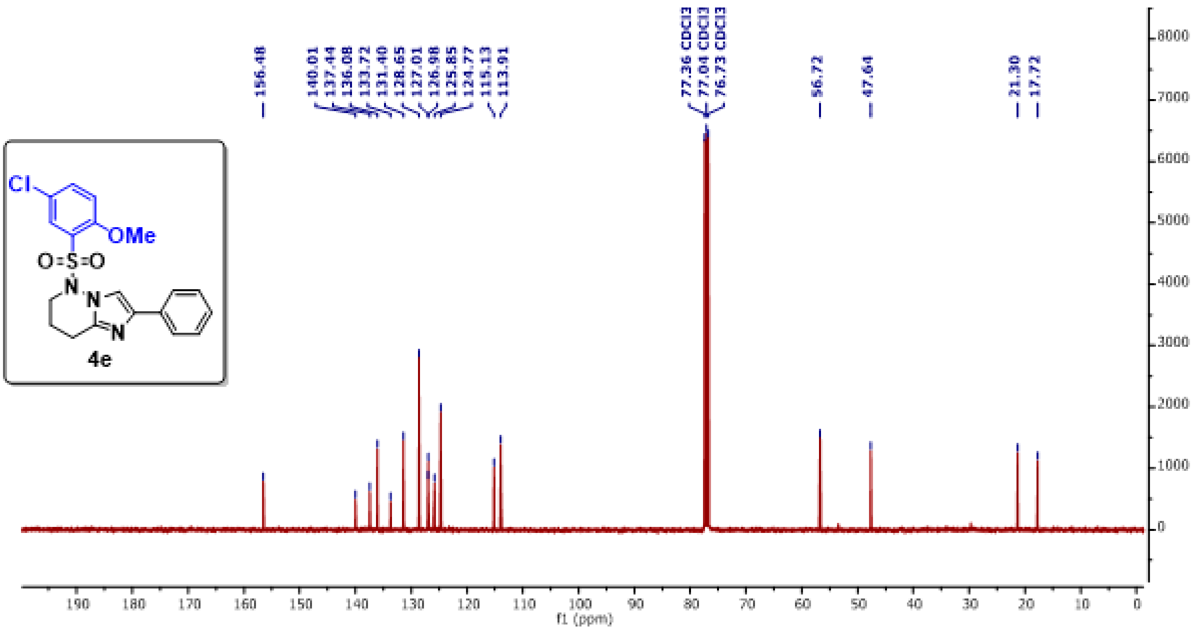

5-((4-(trifluoromethyl)phenyl)sulfonyl)-2-phenyl-5,6,7,8-tetrahydroimidazo[1,2-b]pyridazine (4f)

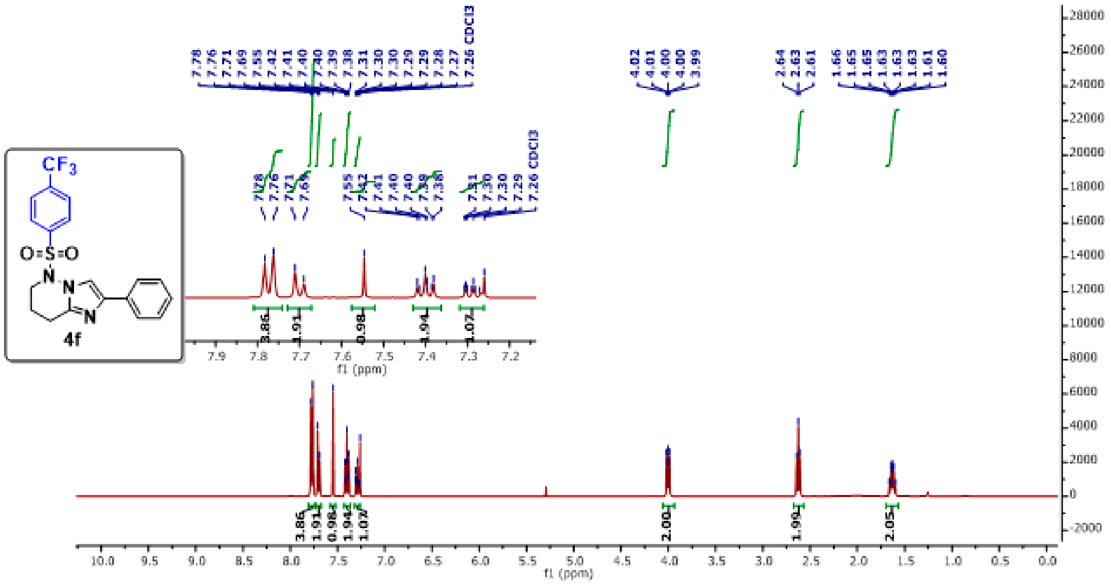

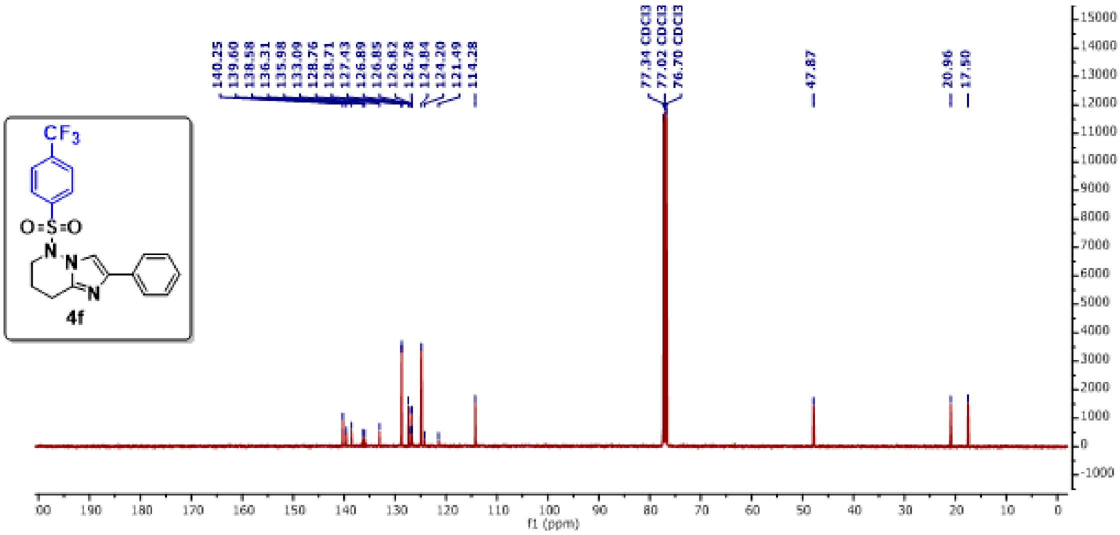

^{19}F NMR spectrum

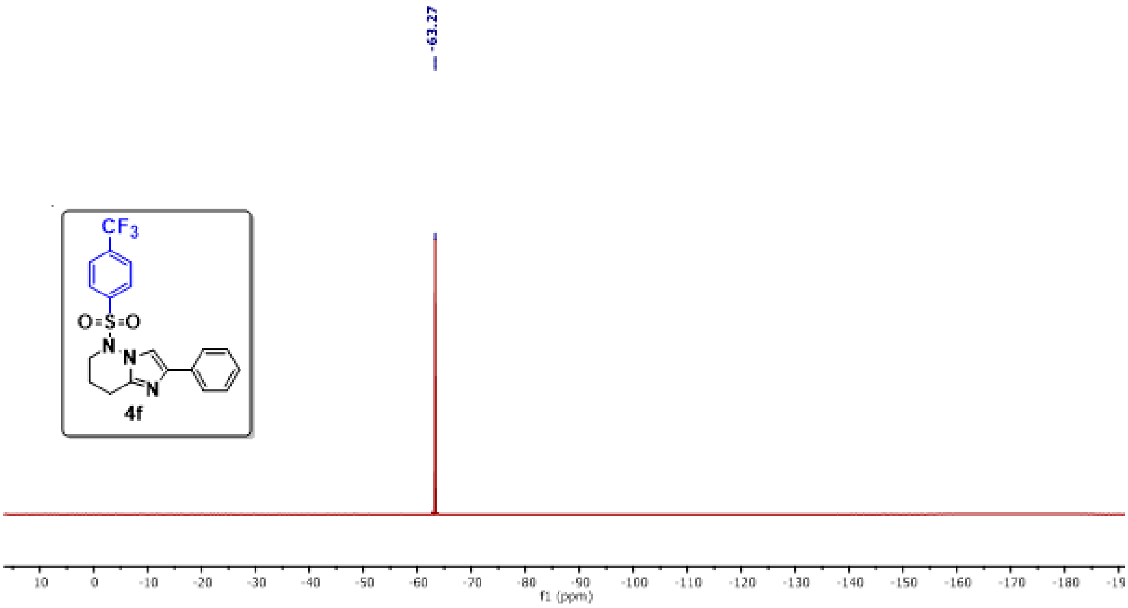

5-(naphthalen-1-ylsulfonyl)-2-phenyl-5,6,7,8-tetrahydroimidazo[1,2-b]pyridazine (4g)

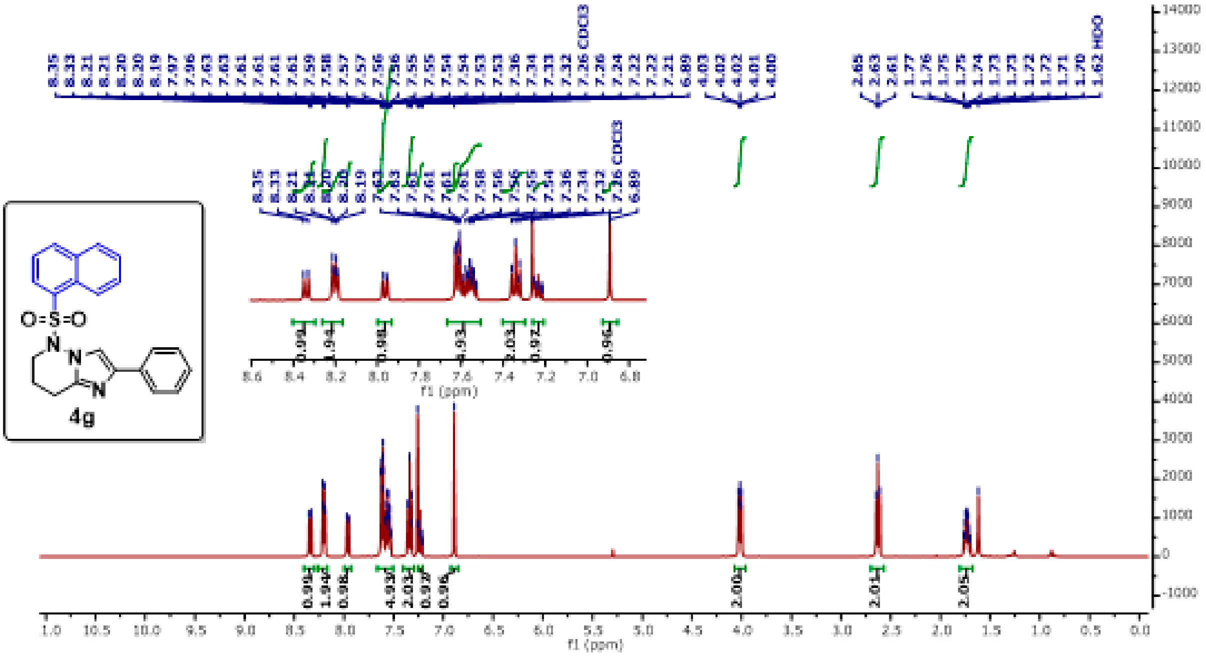

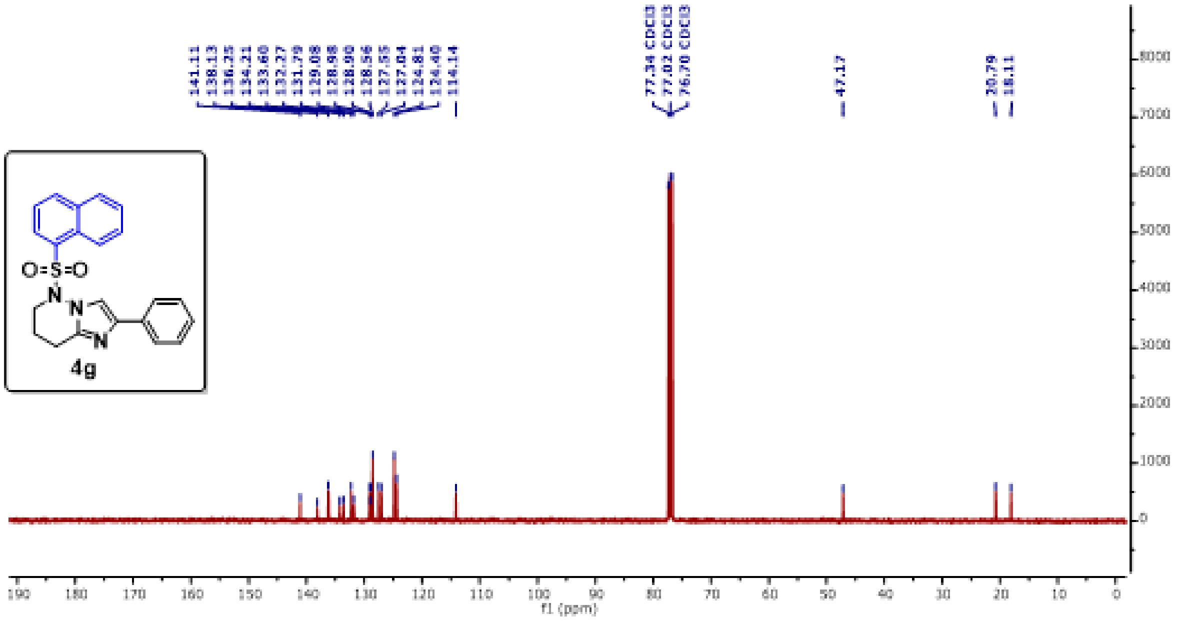

HRMS SPECTR

2-phenyl-5,6,7,8-tetrahydroimidazo[1,2-b]pyridazine (3)

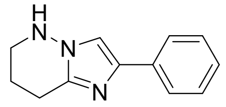

3

Chemical Formula: $C_{12}H_{13}N_3$
Exact Mass: 199,1109

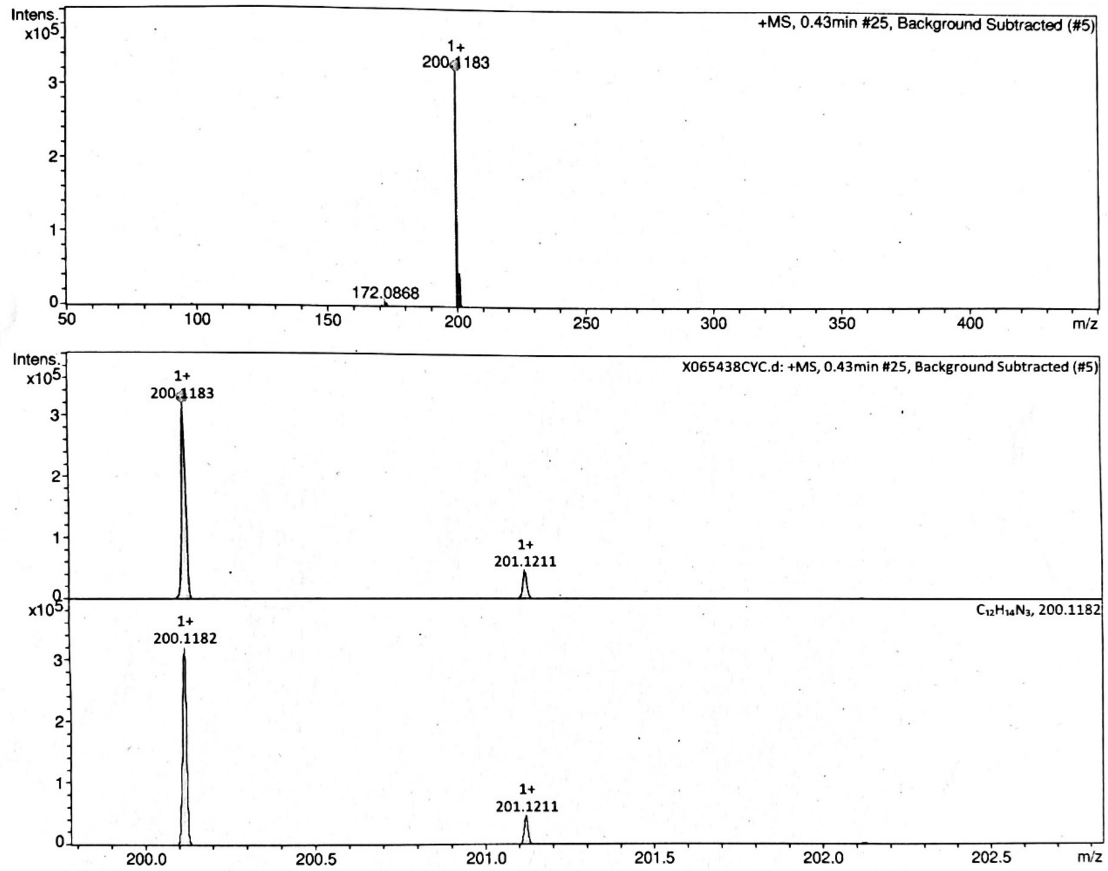

Meas. m/z	z	#	Ion Formula	m/z	err [ppm]	mSigma	rdb	e⁻ Conf
200.118288	1+	1	C12H14N3	200.118224	-0.3	1.7	8.0	even

5-((4-methyphenyl)sulfonyl)-2-phenyl-5,6,7,8-tetrahydroimidazo[1,2-b]pyridazine (4a)

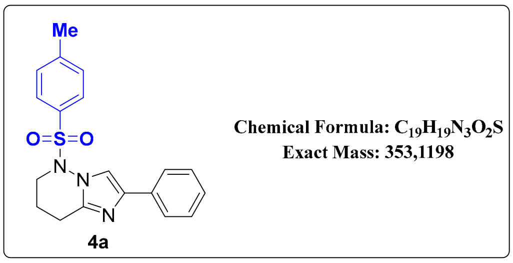

Chemical Formula: $C_{19}H_{19}N_3O_2S$
Exact Mass: 353,1198

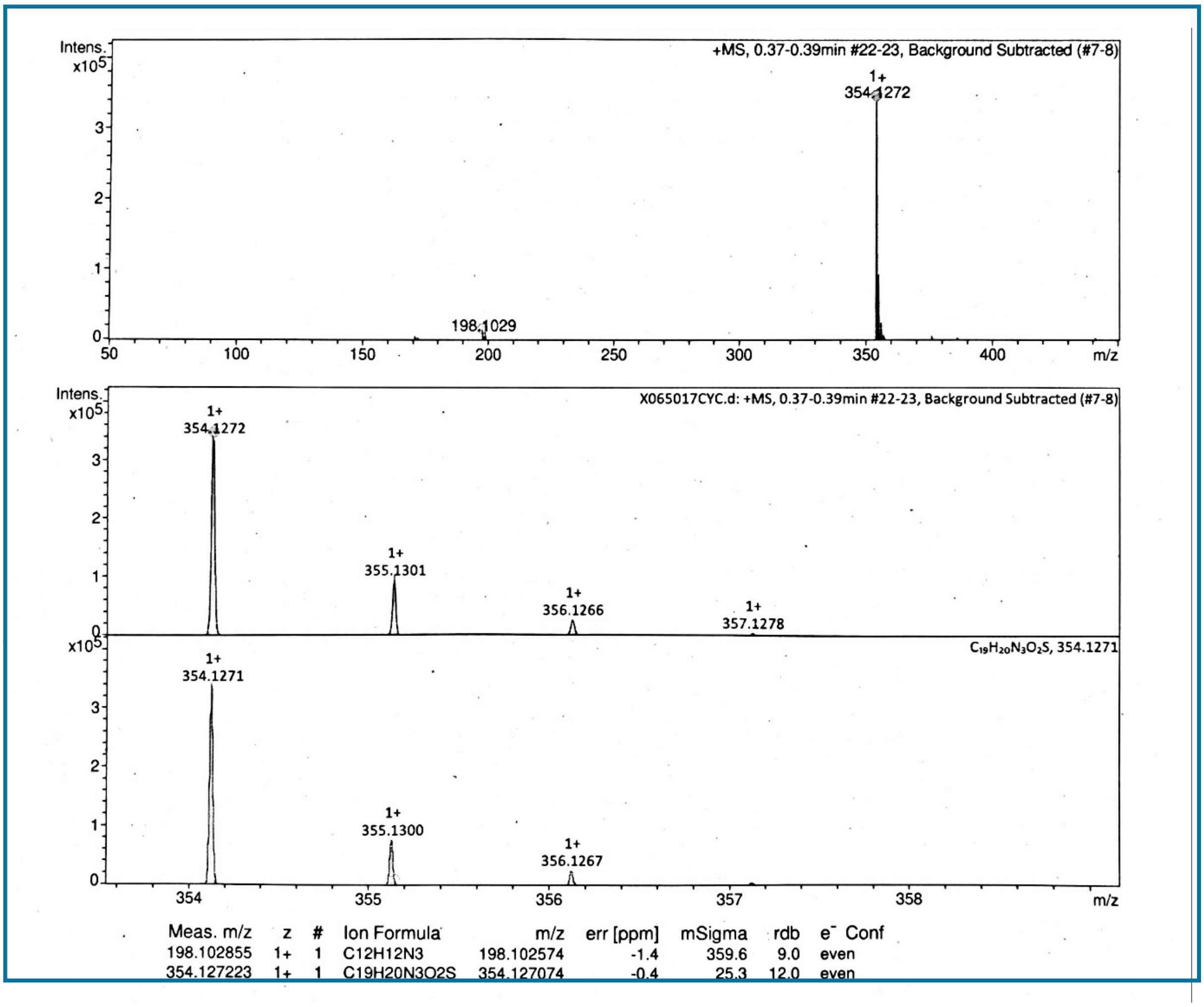

5-((4-methoxyphenyl)sulfonyl)-2-phenyl-5,6,7,8-tetrahydroimidazo[1,2-b]pyridazine (4b)

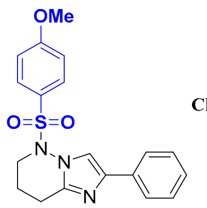

4b

Chemical Formula: $C_{19}H_{19}N_3O_3S$
Exact Mass: 369.11

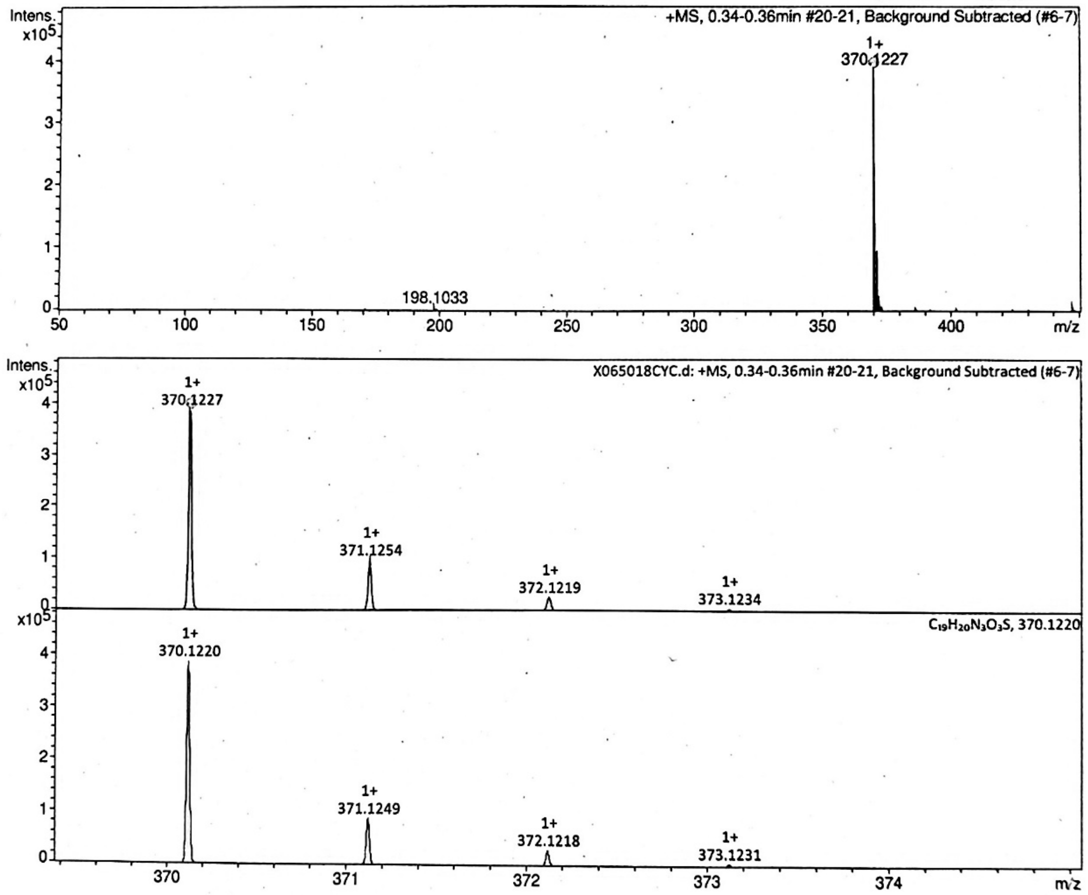

Meas. m/z	z	#	Ion Formula	m/z	err [ppm]	mSigma	rdb	e⁻ Conf
370.122664	1+	1	C19H20N3O3S	370.121989	-1.8	11.9	12.0	even

5-((3,4-dimethoxyphenyl)sulfonyl)-2-phenyl-5,6,7,8-tetrahydroimidazo[1,2-b]pyridazine (4c)

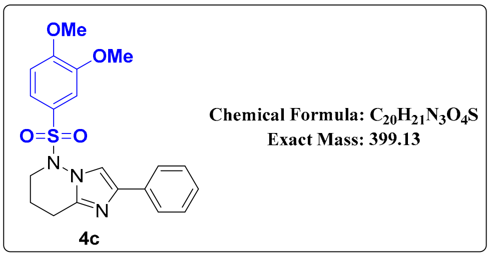

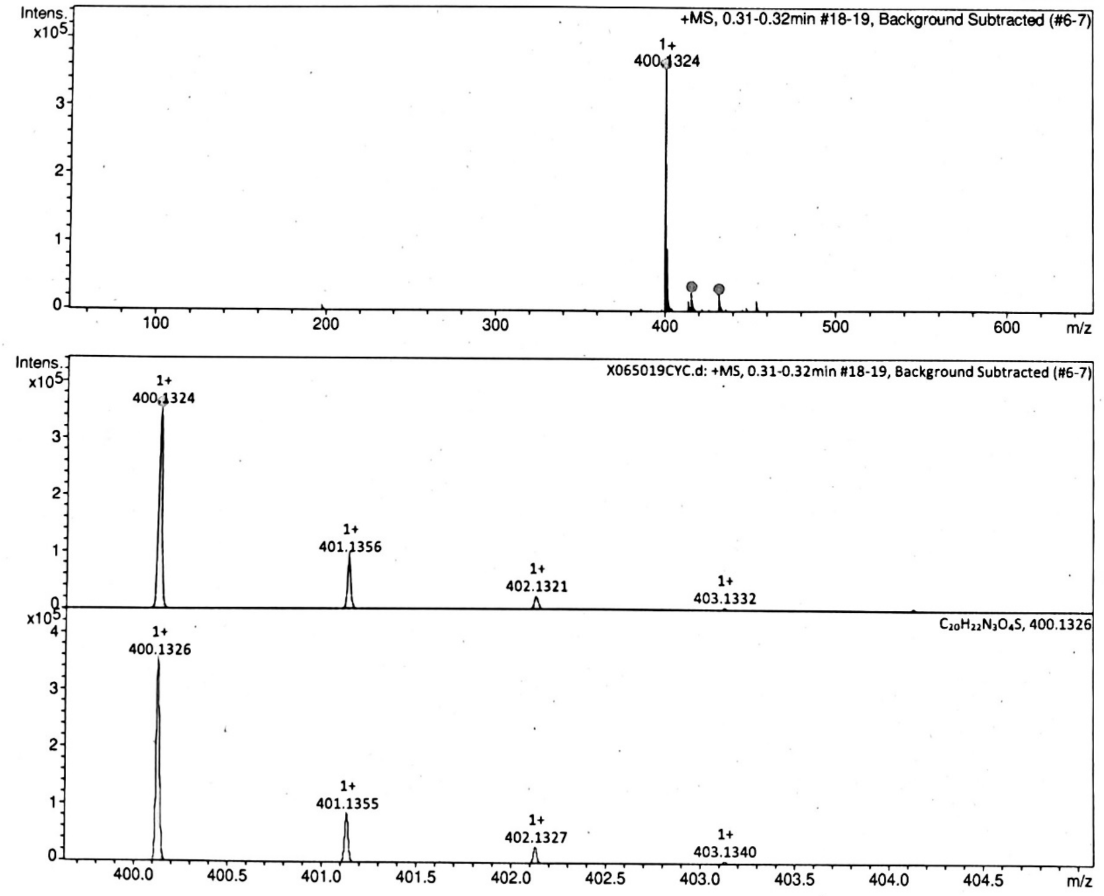

Meas. m/z	z	#	Ion Formula	m/z	err [ppm]	mSigma	rdb	e⁻ Conf
400.132447	1+	1	C20H22N3O4S	400.132554	0.3	12.1	12.0	even
416.127104	1+	1	C20H22N3O5S	416.127468	0.9	14.0	12.0	even
432.122511	1+	1	C20H22N3O6S	432.122383	-0.3	23.5	12.0	even

5-((4-nitrophenyl)sulfonyl)-2-phenyl-5,6,7,8-tetrahydroimidazo[1,2-b] pyridazine (4d)

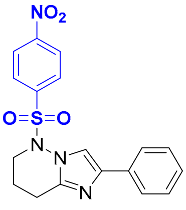

4d

Chemical Formula: $C_{18}H_{16}N_4O_4S$
Exact Mass: 384.09

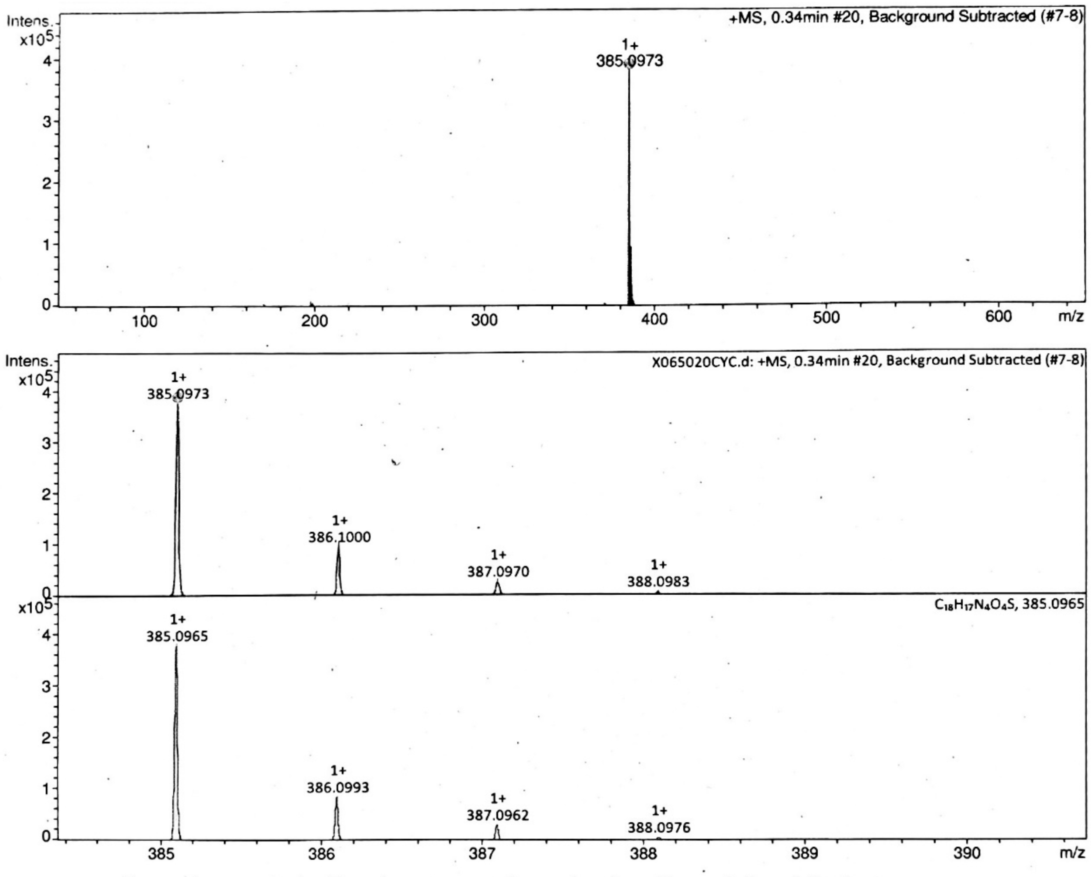

Meas. m/z	z	#	Ion Formula	m/z	err [ppm]	mSigma	rdb	e⁻ Conf
385.097305	1+	1	C18H17N4O4S	385.096502	-2.1	16.0	13.0	even

5-((5-chloro-2-methoxyphenyl)sulfonyl)-2-phenyl-5,6,7 ,8-tetrahydroimidazo[1,2-b]pyridazine (4e)

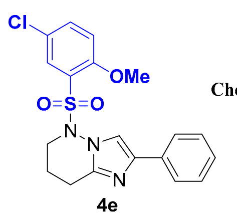

Chemical Formula: $C_{19}H_{18}ClN_3O_3S$
Exact Mass: 403.08

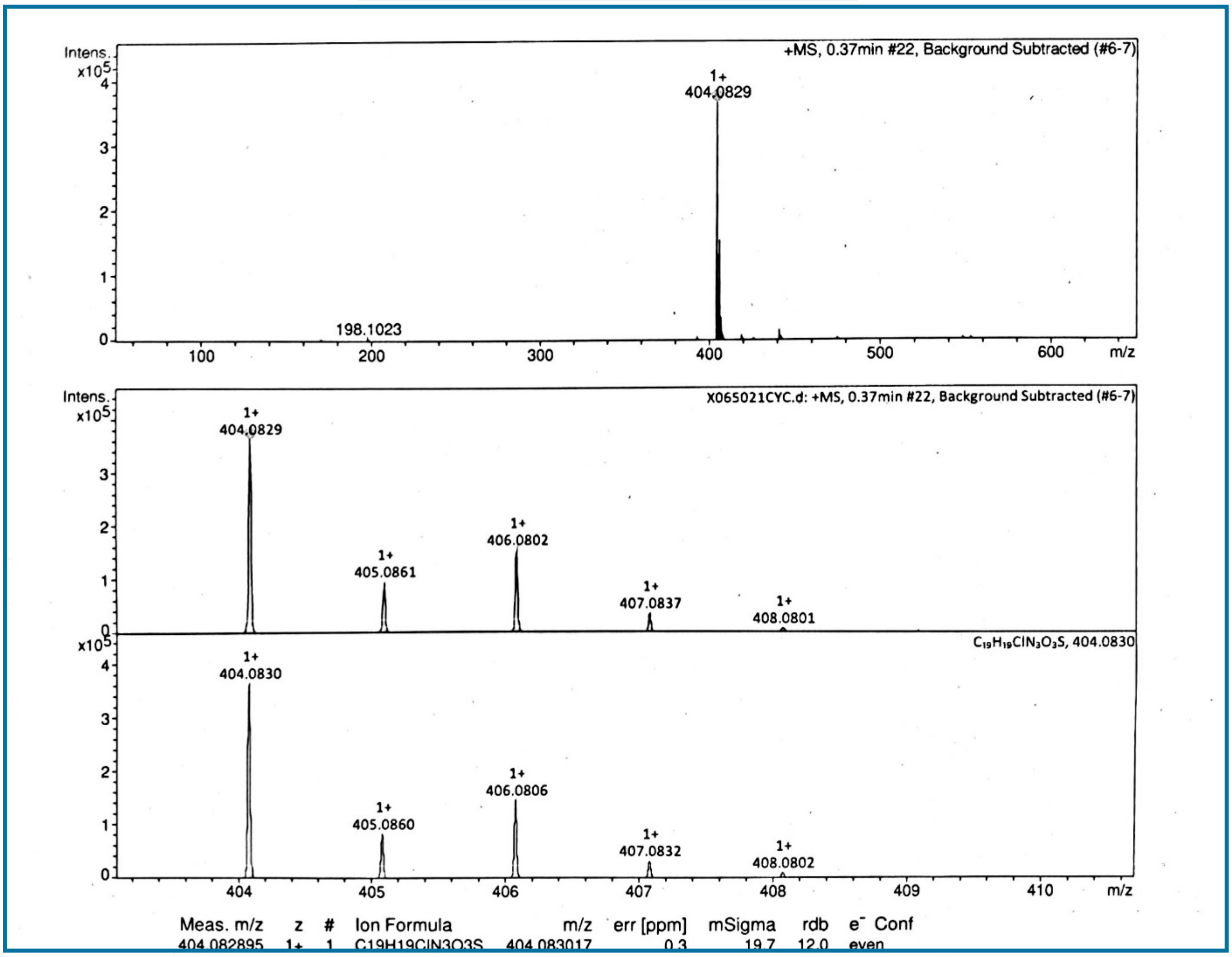

5-((4-(trifluoromethyl)phenyl)sulfonyl)-2-phenyl-5,6,7,8-tetrahydroimidazo[1,2-b]pyridazine (4f)

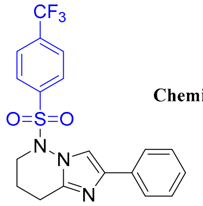

4f

Chemical Formula: $C_{19}H_{16}F_3N_3O_2S$
Exact Mass: 407.09

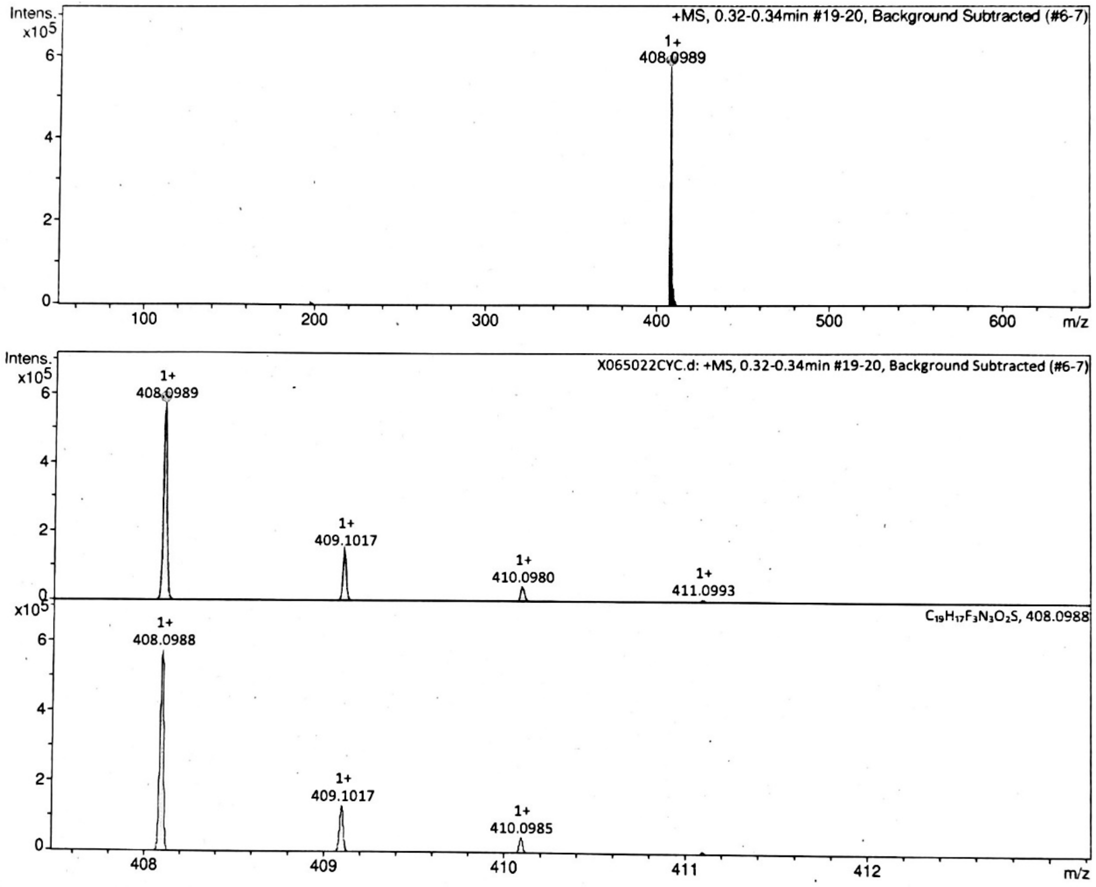

Meas. m/z	z	#	Ion Formula	m/z	err [ppm]	mSigma	rdb	e⁻ Conf
408.098947	1+	1	C19H17F3N3O2S	408.098809	-0.3	14.4	12.0	even

5-(naphthalen-1-ylsulfonyl)-2-phenyl-5,6,7,8-tetrahydroimidazo[1,2-b]pyridazine (4g)

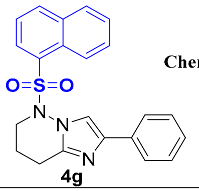

4g

Chemical Formula: $C_{22}H_{19}N_3O_2S$
Exact Mass: 389.12

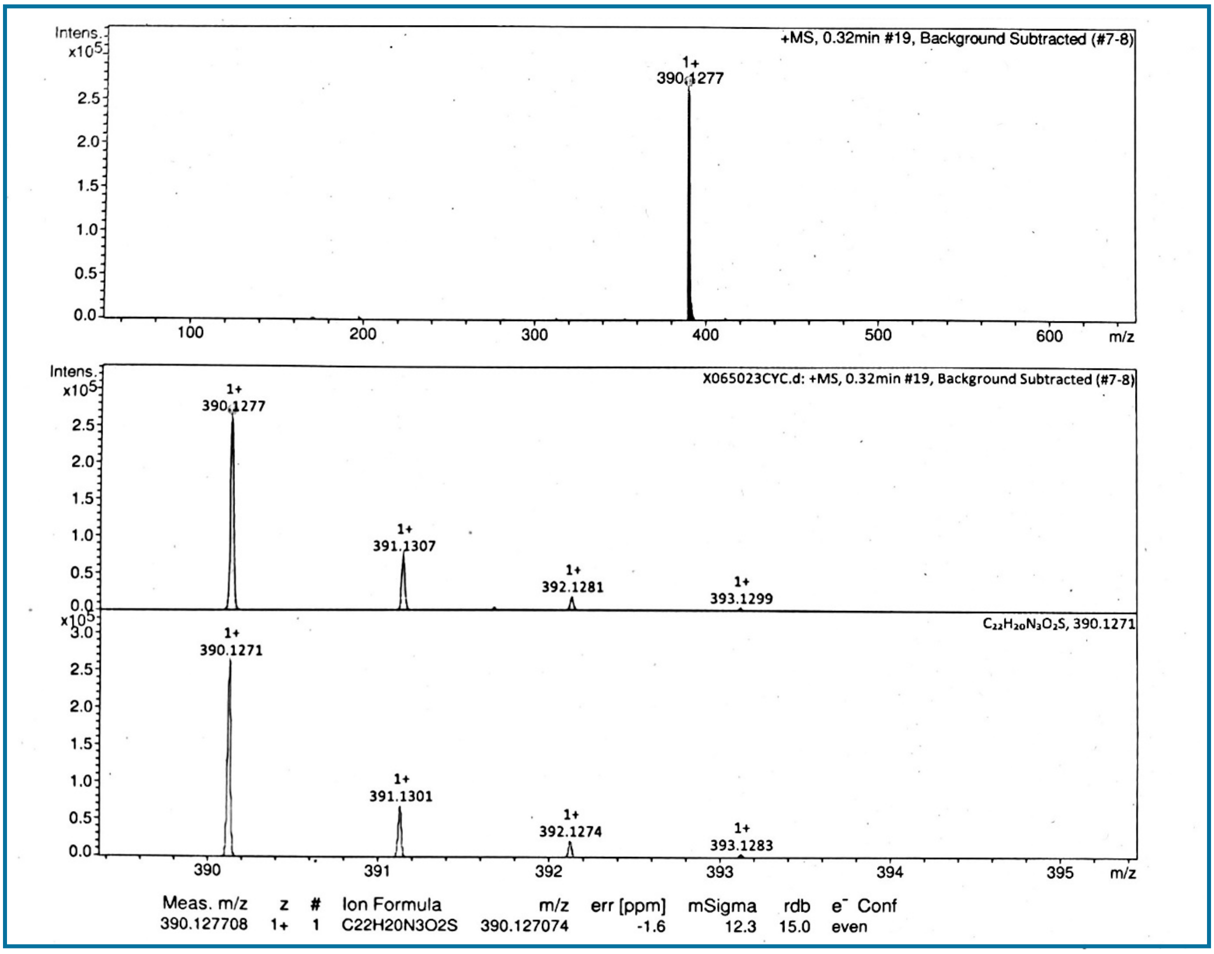

Supplement: Supplementary file 1 [file molecules-27-05238-s001.zip › molecules-1850581-supplementary.pdf]
